# DeorphaNN: Virtual screening of GPCR peptide agonists using AlphaFold-predicted active-state complexes and deep learning embeddings

**DOI:** 10.1016/j.molcel.2026.07.006

**Published:** 2026-07-23

**Authors:** Larissa Ferguson, Sébastien Ouellet, Elke Vandewyer, Christopher Wang, Zaw Wunna, Tony K.Y. Lim, William R. Schafer, Isabel Beets

**Affiliations:** 1 Neurobiology Division, MRC Laboratory of Molecular Biology, Cambridge, UK; 2 Independent Researcher, Ottawa, ON, Canada; 3 Department of Biology, KU Leuven, Leuven, Belgium; 4 Department of Pharmacology, University of Cambridge, Cambridge, UK

## Abstract

Peptide-activated G protein-coupled receptors (GPCRs) regulate physiological processes through interaction with neuropeptides and peptide hormones. Identifying endogenous peptide agonists remains challenging, as peptide-GPCR pairings often follow gene-family relationships that offer limited predictive insight for orphan GPCRs without characterized homologs. Using a dataset of experimentally validated peptide-GPCR interactions from *Caenorhabditis elegans*, we demonstrate that AF-multimer confidence metrics partially discriminate agonist from non-agonist complexes, with improved discrimination using AF-Multistate-derived active-state templates. Feature analysis revealed that AF-multimer’s pair representations outperform single representations, with distinct subregions providing complementary signals. Leveraging these insights, we developed DeorphaNN, a graph neural network integrating active-state GPCR-peptide structural predictions, interatomic interactions, and deep learning embeddings to prioritize putative peptide agonists for experimental screening. DeorphaNN generalized across diverse species, as shown by performance on annelid and human retrospective benchmarks. Experimental validation confirmed predicted agonists for two orphan GPCRs, demonstrating its utility for accelerating peptide-GPCR deorphanization.

## Graphical abstract

**Graphical abstract:**
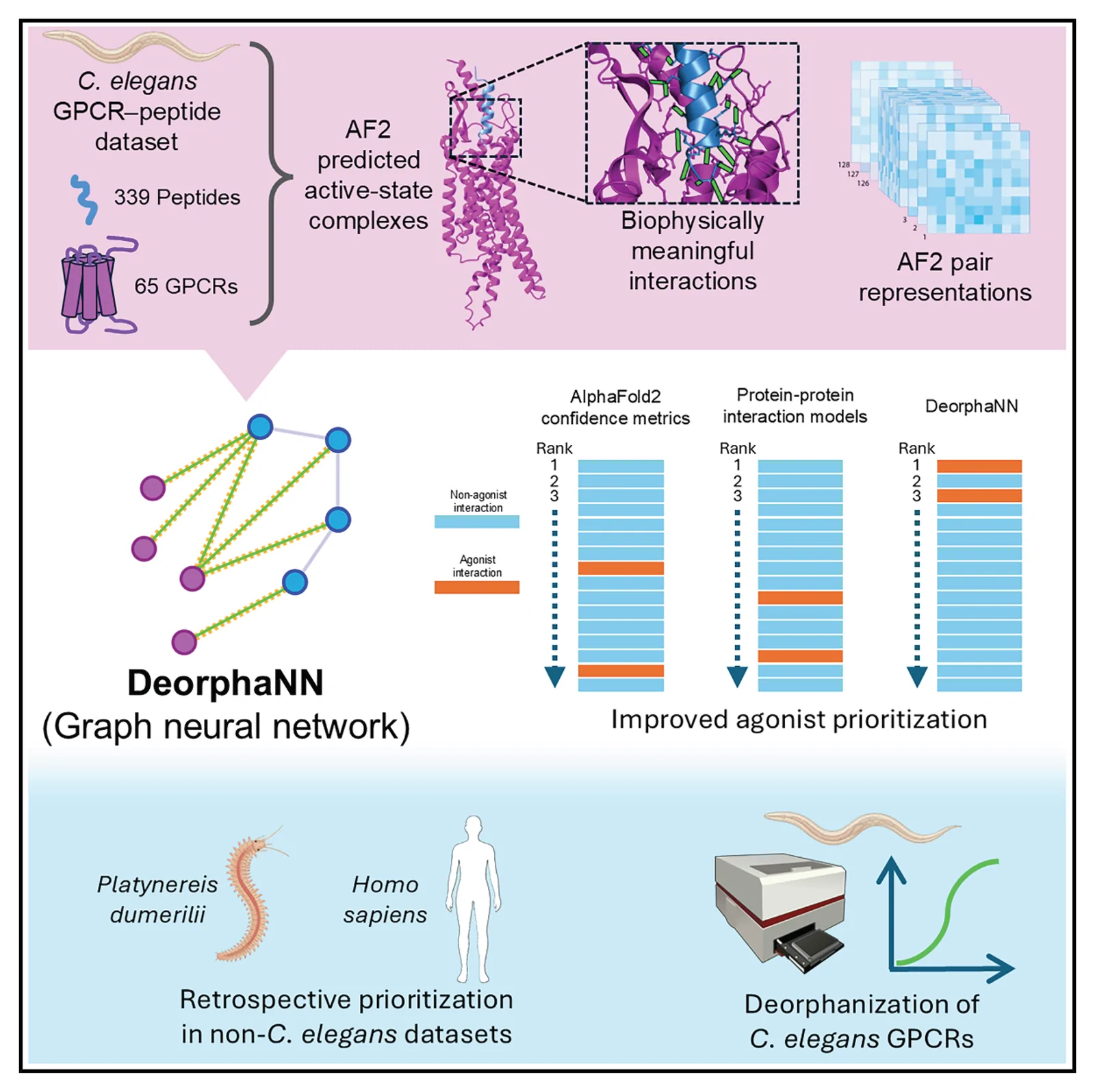

## Introduction

G protein-coupled receptors (GPCRs) are a large and diverse family of membrane proteins critical for cellular signaling, characterized by a seven-transmembrane (TM) domain structure. A key class of GPCR agonists includes small peptides, such as neuropeptides and peptide hormones, which regulate physiological functions ranging from homeostasis and metabolism^1^ to complex behaviors like sleep, mechanosensation, and learning.^2–5^ However, identifying endogenous GPCR-peptide interactions remains a challenge, and many GPCRs are still ‘‘orphan’’ without cognate agonists. Phylogenetic analyses have proven effective for predicting agonists of conserved peptide-activated GPCRs across divergent phyla^6–10^ but are less effective for GPCRs without clear orthologs. Large-scale experimental screens that evaluate libraries of putative bioactive peptides, such as those derived through peptidomics^11^ or computational analysis,^12–14^ have also advanced peptide agonist discovery.^8,10,14,15^ However, these approaches are resource-intensive, as animal genomes often encode dozens to over 100 peptide-activated GPCRs and hundreds of candidate peptides.^8,14–16^

Despite advances in machine learning applications and docking tools for identifying small-molecule ligands for GPCRs,^17–21^ computational tools for *in silico* peptide agonist screening remain underdeveloped. This gap persists partly because peptides—larger, more flexible ligands than small molecules—pose unique challenges for computational modeling.^22^ While docking can predict peptide binding poses,^23^ it often fails to identify functional agonists,^24^ as only specific peptide conformations and binding orientations trigger receptor activation, and most docking algorithms do not capture these dynamic structural changes. This limitation highlights the need for next-generation computational tools specifically designed to address the unique structural and mechanistic complexities of GPCR-peptide agonist interactions.

Advances in protein structure prediction tools are reshaping computational structure-based discovery of protein complexes and interactions. The AlphaFold2 (AF2) algorithm^25^ predicts protein structure by implicitly learning biophysical relationships between residues through a combination of coevolutionary data and deep learning techniques.^26^ These biophysical properties, encoded in AF2’s protein representations, enable applications beyond structural modeling.^27–29^ AF-multimer,^30^ an extension of AF2, allows accurate prediction of multimeric complexes, including peptide-protein interactions.^31,32^ At the same time, system-wide experimental screens are expanding our knowledge of GPCR-peptide interactions across diverse animal species. These advances—generalizable AF2-derived protein representations, AF-multimer’s peptide-protein docking capabilities, and high-quality GPCR-peptide interaction data—provide a foundation for developing computational methods to predict peptide agonists for GPCRs.

In this study, we harness AF-multimer-derived structural predictions and hidden-layer protein representations to develop computational methods for discriminating agonist and non-agonist GPCR-peptide interactions. We build this method on an experimentally validated dataset of GPCR-peptide agonist and non-agonist pairs that we constructed previously in *Caeno-rhabditis elegans* by screening >55,000 GPCR-peptide interactions.^8^ Using this dataset, we evaluate AF-multimer confidence metrics as indicators of agonist activity, revealing their modest discriminative capability. This capability is enhanced when active-state GPCR-peptide complexes—modeled using templates derived from AF-Multistate^33^—are utilized to align receptors with activation-associated binding conformations. Next, we analyze AF-multimer’s hidden layer protein representations and find that pair representations outperform single representations in distinguishing agonists. We find that subregions within the pair representations contribute distinct, complementary predictive signals, highlighting the importance of multi-modal feature integration.

Building on these insights, we developed DeorphaNN, a graph neural network (GNN) that integrates AF-multimer-derived structural predictions, interatomic interactions, and protein representations to prioritize putative agonist interactions. We demonstrate that this pipeline generalizes across datasets derived from different species, including *Platynereis* and human retrospective benchmarks. Experimental validation of peptide agonists prioritized by DeorphaNN identified endogenous peptide ligands of two orphan *C. elegans* GPCRs, demonstrating DeorphaNN’s potential for enhancing conventional screening and accelerating the deorphanization of peptide-activated GPCRs.

## Results

### Dataset preprocessing

**Figure 1:**
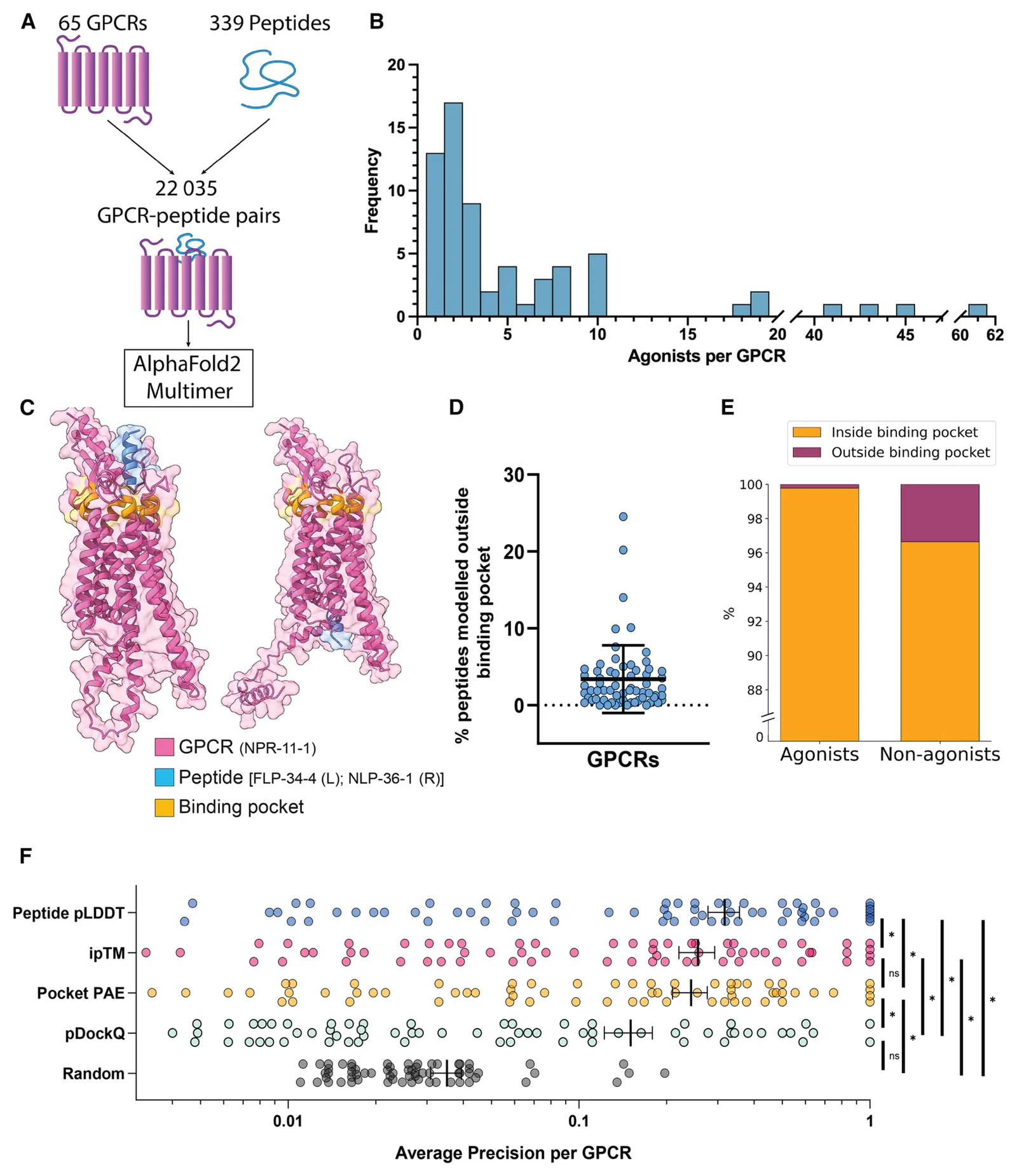
AF-multimer structures and confidence metrics exhibit limited ability to discriminate agonist from non-agonist GPCR-peptide interactions (A) 65 GPCRs and 339 endogenous peptides (22 035 GPCR-peptide pairs) from *C. elegans* were modeled using AF-multimer. (B) Frequency distribution of the number of agonists per GPCR in the dataset. Median = 3. (C) Example AF-multimer predicted structures. Left: FLP-34-4, an agonist for the GPCR, NPR-11-1, is modeled in the orthosteric binding pocket. Right: NLP-36-1, a non-agonist for NPR-11-1, is modeled outside of the binding pocket. The GPCR is shown in pink, binding pocket residues (defined as ±5 residues from the extracellular-membrane boundary) in yellow, and peptides in blue. (D) The percentage of peptides modeled outside the binding pocket for each GPCR ranges from 0%–24.5%, with a mean of 3.4% (±4.4% SD). (E) 99.8% of agonists are modeled within the GPCR binding pocket, compared with 96.6% of non-agonists. (F) Peptide pLDDT (mAP = 0.32 ± 0.039) significantly outperforms ipTM (mAP = 0.26 ± 0.036), pocket PAE (mAP = 0.24 ± 0.033), pDockQ (mAP = 0.15 ± 0.028), and random ranking (mAP = 0.04 ± 0.004) in distinguishing agonists from non-agonists. pDockQ is the only metric that does not differ significantly from random ranking (Friedman test with false discovery rate [FDR]-corrected multiple comparisons using the method of Benjamini, Krieger, and Yekutieli, *q < 0.05). The *x* axis is plotted on a log10 scale. Error bars indicate mean ± SEM.

We leveraged a previously published dataset that employed a high-throughput Chinese hamster ovary (CHO) Gα_16_-mediated calcium signaling assay to screen peptide agonist activity across 161 putative peptide-activated GPCRs from *C. elegans*.^8^ We only included GPCRs that showed concentration-dependent activation by at least one peptide, as a lack of activity could have been due to factors such as non-functional expression, misfolding, or failure to couple with Gα_16_, rather than the absence of a true agonist. The dataset primarily consisted of class A GPCRs, with only two class B GPCRs (SEB-3-1 and PDFR-1-1). Following filtering (see STAR Methods), our final dataset contained 65 GPCRs (derived from 55 genes) and 339 unique peptides (derived from 93 genes) (Figure 1A). Combining each GPCR with each peptide (‘‘GPCR-peptide complex’’) yielded 20,035 pairs, 457 of which were experimentally confirmed agonist pairs.^8^ The median number of agonists per GPCR in the dataset was 3 and ranged from 1 to 61 (Figure 1B). For 38 of the 65 GPCRs, agonist peptides were derived from a single peptide-encoding gene, with the specific gene varying across receptors (Figure S1A). Peptides ranged in size from 3 residues to 37 residues, with a median length of 11 residues (Figure S1B). The names and amino acid sequences of all GPCRs and peptides in the training dataset are provided in Data S1.

### AF-multimer positions peptides in the GPCR binding pocket with minimal discrimination

We modeled each GPCR-peptide complex using AF-multimer to evaluate whether the structural outputs could distinguish agonists from non-agonists. As peptide agonists typically interact with the helical cavity (‘‘orthosteric binding pocket’’) and extracellular loops of GPCRs in experimentally determined structures,^34^ we hypothesized that AF-multimer would predominantly model agonist peptides in a similar manner, while non-agonist peptides might be modeled in less physiologically relevant locations (Figure 1C). To investigate this across diverse GPCR structures, we defined the putative GPCR binding pocket (see STAR Methods) and computed the minimum peptide-to-pocket residue distance to classify peptides as being ‘‘inside’’ or ‘‘outside’’ the GPCR binding pocket. On average, ~5% of peptides per GPCR were predicted to be further than 12.5 Å from the nearest binding pocket residue, though this varied by GPCR (Figure 1D). The GPCR, NPR-40-1, had the highest number of peptides predicted outside the binding pocket (24.5%). Across the dataset, only one agonist peptide was modeled outside of the binding pocket (GPCR: DMSR-1-1; peptide: FLP-11-4), compared with ~3.4% of non-agonist peptides (Figure 1E). Due to the prevalence of peptides positioned in the binding pocket, this feature alone does not provide a reliable basis for distinguishing between agonist and non-agonist GPCR-peptide complexes.

### AF-multimer confidence metrics partially discriminate peptide agonists from non-agonists

We next evaluated the ability of AF-multimer confidence metrics to discriminate peptide agonists from non-agonists. The interface pTM (ipTM) is a metric that assesses the accuracy of the protein-protein interface in the predicted multimeric complex and is reported as a single number per multimer, ranging from 0 (low accuracy) to 1 (high accuracy).^30^ The predicted local distance difference test (pLDDT) is a measure of confidence, output as a per-residue score ranging from 0 (low confidence) to 100 (high confidence). Since the TM regions of GPCRs are typically predicted with high confidence by AF2, we focused on confidence metrics of the peptide, averaging across peptide residues to yield a single aggregated ‘‘peptide pLDDT’’ score for each GPCR-peptide complex. The predicted aligned error (PAE) quantifies the expected positional error of each residue in a pairwise alignment with every other residue, output as a 2D array with values ranging from 0 to 31.75 Å. Per-residue PAE scores for the peptide aligned on putative GPCR binding pocket residues were averaged to yield the ‘‘pocket PAE’’ score. We also evaluated the predicted DockQ (pDockQ) score, which estimates the reliability of a complex by integrating pLDDT and the number of interface contacts between protein chains.^35^ In general, agonist GPCR-peptide complexes score better than non-agonist complexes across all metrics (Figures S1C–S1F).

We evaluated the predictive capability of each metric using mean average precision (mAP) (see STAR Methods for calculation). In brief, average precision (AP) measures how agonists rank relative to non-agonists for each GPCR, with a value of 1 indicating that all agonists for that GPCR are ranked in the highest positions. The mAP is an average of AP scores across all receptors. This approach avoids disproportionately assigning higher weight to receptors with many agonists and reduces sensitivity to the class imbalance inherent in the dataset. Furthermore, AP is higher when agonists are ranked highly, better reflecting practical screening scenarios in which only a limited number of top-scoring candidates are selected for experimental validation. Analysis of the AP for AF-multimer confidence metrics revealed their ability to discriminate agonists from non-agonists, while pDockQ did not differ significantly from random ranking (Figure 1F). Receiver operating characteristic (ROC) and precision-recall (PR) curves are provided in Figures S1G and S1H. Peptide pLDDT significantly outperformed all other metrics.

### Active-state GPCR-peptide complexes enhance the ability of peptide pLDDT to discriminate agonists

AF2 is inherently biased toward modeling GPCRs in an inactive conformation.^33^ As agonist binding stabilizes the active conformation of GPCRs,^36^ we hypothesized that biasing the GPCR in GPCR-peptide complexes toward an active conformation would improve the prediction confidence for agonists.

**Figure 2:**
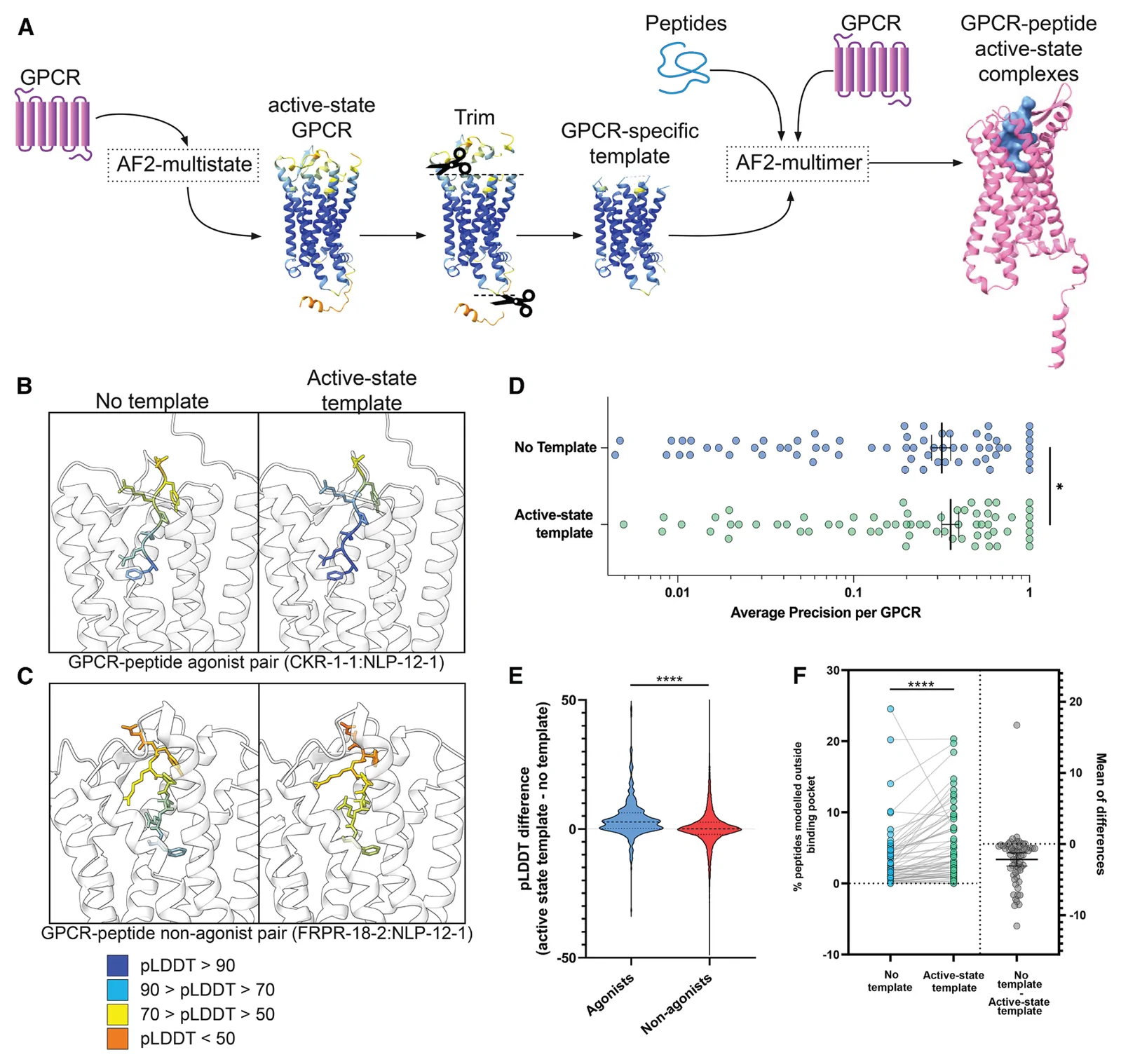
Active-state templates enhance AF-multimer’s discrimination of agonist and non-agonist GPCR-peptide interactions (A) Predicted active-state structures were generated using AF-Multistate. Extracellular regions and low-confidence intracellular regions (pLDDT < 70) were trimmed and used as templates in AF-multimer to model GPCR-peptide complexes. (B and C) Representative agonist (CKR-1-1 with NLP-12-1) and non-agonist (FRPR-18-2 with NLP-12-1) complexes modeled without and with an active-state template. Active-state templates improved peptide pLDDT for the agonist complex but not the non-agonist complex. (D) Average precision (AP) of agonist peptides ranked by peptide pLDDT modeled in the absence and presence of active-state templates. Active-state templates (mAP = 0.36 ± 0.039) significantly improved AP compared with modeling without templates (mAP = 0.32 ± 0.039) (the no-template condition is identical to Figure 1F; Wilcoxon signed-rank test, *p < 0.05). Error bars indicate mean ± SEM. The *x* axis is plotted on a log10 scale. (E) Change in peptide pLDDT with active-state templates was significantly greater for agonists compared with non-agonists (Welch’s *t* test, ****p < 0.0001). Lines represent the median and interquartile ranges. (F) Active-state templates increased the percentage of peptides modeled outside the GPCR binding pocket (mean ± SEM: 5.5% ± 0.6% vs. 3.4% ± 0.6% without templates; paired *t* test, ****p < 0.0001).

To model active-state GPCR-peptide complexes, we generated predicted active-state GPCR structures using AF-Multistate^33^ and refined them for use as templates (Figure 2A; STAR Methods). First, we trimmed extracellular regions from the template, as these domains mediate ligand binding.^34^ This allows AF-multimer to model peptide interactions without interference from predefined extracellular structures. We preserved the TM helices, as conformational shifts to the active state are driven by their displacement.^37–39^ Next, we removed low-confidence residues in the intracellular domain (involved in G protein interactions), preventing these regions from distorting structural predictions. By identifying extracellular, intracellular, and TM regions using DeepTMHMM and low-confidence residues using pLDDT, this standardized method can be used to generate a predicted active-state template for any GPCR.

Active-state templates enhance discrimination of agonist and non-agonist GPCR-peptide interactions. Figure 2B illustrates an example where modeling a GPCR-peptide agonist complex with an active-state template increases peptide pLDDT. By contrast, Figure 2C shows the same peptide modeled with a GPCR for which it is not an agonist. Here, the active-state template does not alter peptide pLDDT. Active-state templates increase the predictive capability of peptide pLDDT compared with template-free modeling (Figure 2D; Figures S1I and S1J) by increasing peptide pLDDT for agonists relative to non-agonists (Figure 2E; Figure S1K). A greater proportion of non-agonist peptides are positioned outside of the binding pocket when using active-state templates (Figure 2F). By contrast, active-state templates did not alter the number of agonist peptides modeled outside of the binding pocket—as with no-template models, only a single agonist peptide was modeled outside the pocket (GPCR: DMSR-1-1; peptide: FLP-11-4). These findings demonstrate that active-state GPCR templates improve discrimination between agonist and non-agonist complexes. We therefore employed active-state templates for all subsequent analyses.

### Pair representations outperform single representations in discriminating GPCR-peptide agonists

**Figure 3:**
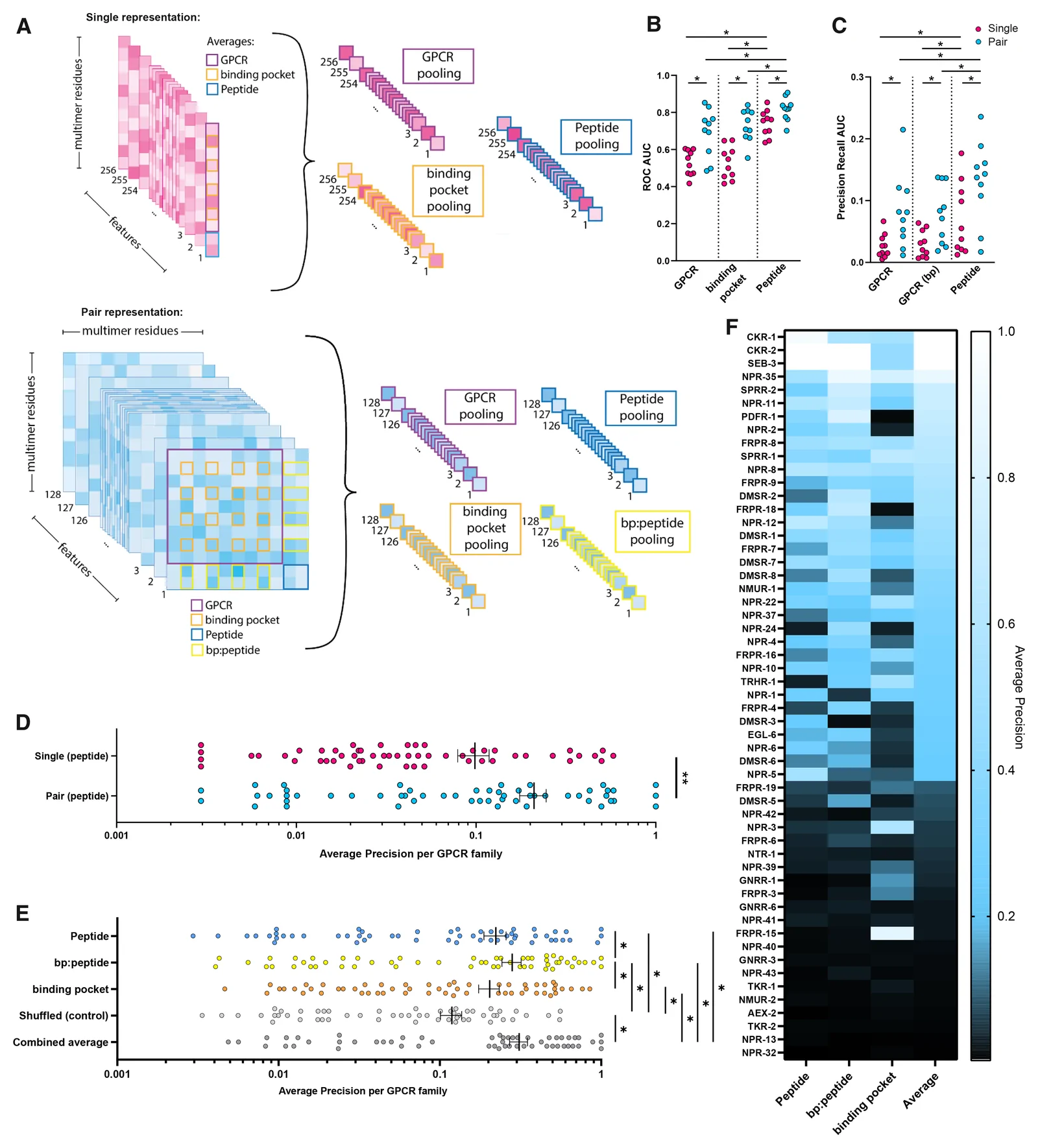
Subregions of AF-multimer pair representations provide information for classifying agonist and non-agonist peptides (A) Schematic illustration of feature extraction from AF-multimer representations. Single representations (ℝ^{*n*×128}^) and pair representations (ℝ^{*n*×*n*×128}^) were processed by aggregating features via local pooling over predefined subregions: peptide residues (blue outlines), GPCR residues (purple outlines), binding pocket residues (orange outlines), and the pairwise interaction of binding pocket and peptide residues (‘‘bp:peptide’’; yellow outlines). All analyses in (B)–(F) were performed using random forest models with 10-fold cross-validation and GPCRs clustered by phylogenetic similarity. All *x* axes are shown on a log10 scale. Data are presented as mean ± SEM. (B and C) ROC-AUC and PR-AUC for models trained on single and pair representations using different subregion-based pooling schemes. Pair representations significantly outperform single representations, and peptide subregions outperform GPCR and binding pocket subregions. Statistical significance was assessed by two-way repeated measures ANOVA with Geisser-Greenhouse correction and post-hoc multiple comparisons carried out by FDR correction using the method of Benjamini, Krieger, and Yekutieli (*q < 0.05). (ROC-AUC, F (1.415, 12.73) = 5.691, *p* = 0.0246; PR-AUC, F (1.715, 15.43) = 8.550, *p* = 0.0042. (D) AP for models trained on peptide single and pair representations. Pair representations (mAP = 0.21 ± 0.035) significantly outperform single representations (mAP = 0.10 ± 0.020) (Wilcoxon matched-pairs signed-rank test; **p < 0.01). (E) AP averaged across five models trained on different subregions of the pair representations along with a combined average and a shuffled control (peptide and bp shuffled, bp:peptide fixed). The combined average (mAP = 0.31 ± 0.039) significantly outperforms individual regions (peptide mAP = 0.22 ± 0.035, bp:peptide mAP = 0.28 ± 0.038, bp mAP = 0.21 ± 0.030) and the shuffled control (mAP = 0.12 ± 0.018). Statistical significance was assessed using a Friedman test with FDR-corrected multiple comparisons (Benjamini, Krieger, and Yekutieli; *q < 0.05). (F) Heatmap of AP values from (E) illustrates that different GPCR gene families benefit from features provided by different subregions.

When predicting 3D protein structure, AF2 generates ‘‘single’’ and ‘‘pair’’ representations that are transformed and updated in the EvoFormer block of the algorithm.^25^ Each single representation is a matrix in ℝ^{*n*×256}^, where *n* is the number of residues in the GPCR-peptide complex. Similarly, each pair representation is a tensor in ℝ^{*n*××*n*××128}^, providing pairwise residue-level relationships (Figure 3A).

We applied local pooling to residue subregions in each representation obtained from AF2 run with active-state templates to identify features useful for classifying GPCR-peptide agonists. For single representations, we averaged residue regions corresponding to the GPCR, the binding pocket, or the peptide, producing three different 256-dimensional feature vectors. Similarly, for pair representations, we averaged residue embeddings involving the same regions and additionally the pairwise intersection of the binding pocket and peptide residues (‘‘bp:peptide’’), yielding four distinct 128-dimensional feature vectors.

To evaluate the relative importance of features across subregions of the single and pair representations, we performed feature importance analysis using random forest classifiers. In this context, the classifiers are not optimized for predictive accuracy but rather serve as a relative measure of feature importance for comparing how the different subregions support agonist/ non-agonist discrimination. To ensure that closely related GPCRs were not split across cross-validation folds, we performed a phylogenetic analysis on the GPCR dataset (Figure S2). Full-length GPCR sequences were aligned, trimmed, and clustered into groups of closely related receptors (Data S1). These clusters were used to define cross-validation splits, minimizing overlap in training and test splits. Additionally, the training dataset was stratified by agonist class to help reduce class imbalance. Our analysis demonstrates that features derived from the peptide subregions (from both single and pair representations) outperform those from GPCR and binding pocket subregions as measured by ROC (Figure 3B; Figures S3A and 3C) and PR (Figure 3C; Figures S3B and 3D) area under the curve (AUC). Notably, pair representations significantly outperformed single representations across all pooling strategies. Because metrics like ROC-AUC and PR-AUC disproportionately weight promiscuous GPCRs, we computed AP per GPCR gene family to ensure each GPCR gene contributes equally regardless of agonist count or the number of GPCR isoforms derived from that gene. We found that pair representations achieve significantly higher mAP than single representations (Figure 3D). Their performance remains unchanged when cross-validation is performed using peptides clustered by similarity, as well as leave-one-group-out cross-validation with GPCR or peptide gene groups (Figures S3E–S3G), indicating that performance is not due to the model encountering similar peptides or GPCRs in both training and test sets.

### Distinct subregions of pair representations contribute differently to agonist prioritization

To further investigate which pair representation subregions drive agonist prioritization, we trained random forest models on three key subregions: (1) the bp:peptide subregion (pairwise interaction between the binding pocket and the peptide residues), (2) the peptide subregion, and (3) the binding pocket subregion.

Models trained on the binding pocket subregion exhibited significantly lower AP scores compared with those trained on the bp:peptide or peptide subregions (Figure 3E). However, despite lower scores, binding pocket-trained models were able to prioritize certain agonists that eluded models trained on other subregions (Figure 3F), such as for the GPCRs FRPR-15, GNRR-1, and NPR-3. Similarly, peptide-trained models showed superior performance for some GPCRs, while bp:peptide-trained models were more effective for others. The combined average across the three subregion models improved performance beyond that of any individual model, whereas a shuffled control showed no such improvement (Figure 3E). Together, this suggests that different subregions capture complementary aspects of GPCR-peptide interactions.

### Integration of multimodal GPCR-peptide features for prioritizing agonist interactions

**Figure 4:**
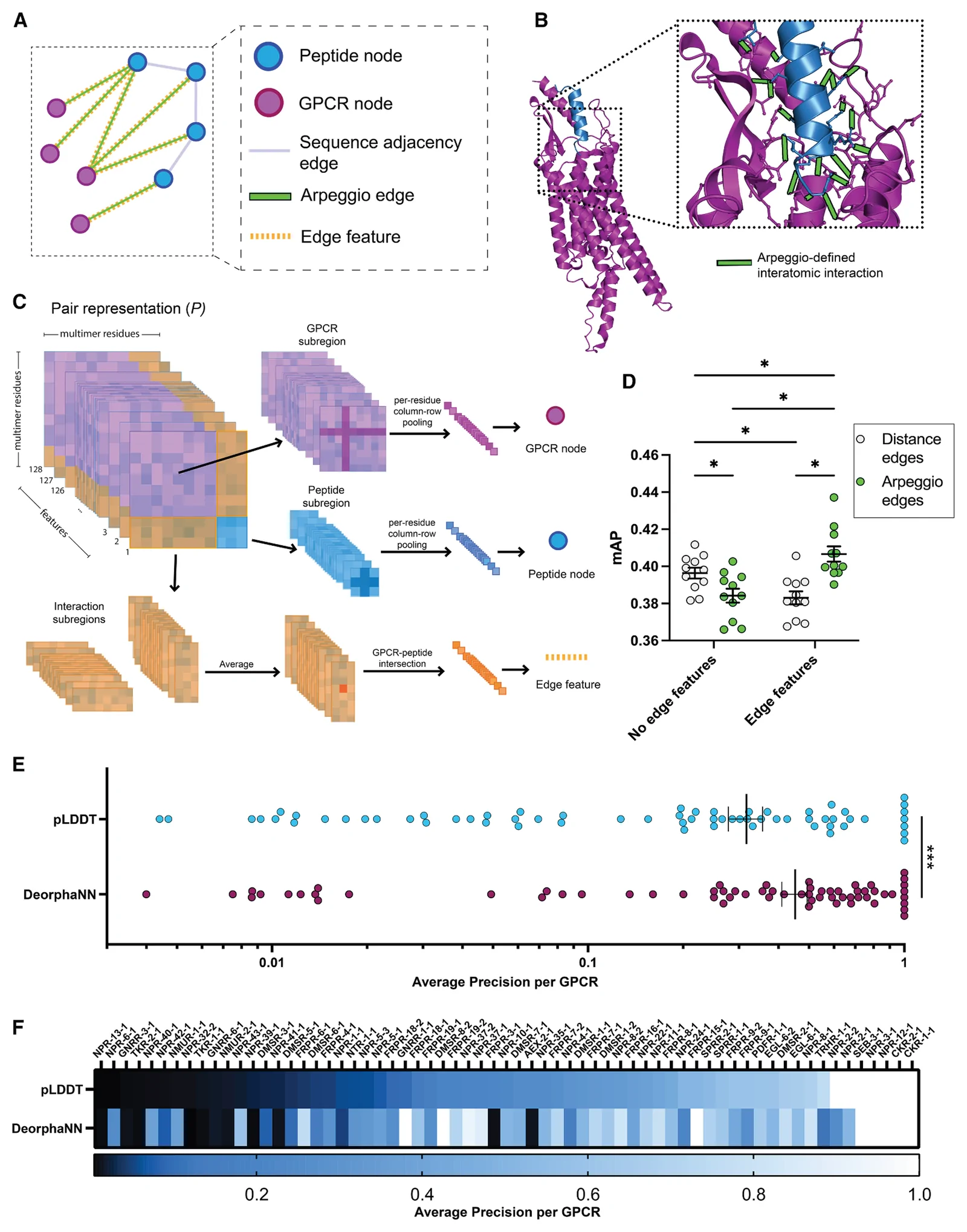
DeorphaNN integrates predicted active-state complexes, interatomic interactions, and protein representations to prioritize GPCR-peptide agonists (A) Graph representation of GPCR-peptide complexes. Nodes correspond to all peptide residues and a subset of GPCR residues directly interacting with peptides, defined using Arpeggio. Intra-protein edges are defined by sequence adjacency, while inter-chain edges are defined using Arpeggio. (B) AF-multimer predicted GPCR-peptide complex showing inter-chain interactions identified by Arpeggio. (C) Construction of node and edge embeddings from AF-multimer pair representations. Pair representations are split into GPCR, peptide, and interaction subregions. Residue-level pooling over GPCR and peptide subregions generates node embeddings, while interaction edges are annotated with pairwise interaction embeddings intra-chain edges are assigned a uniform feature vector equal to the mean feature vector of Arpeggio edges. (D) Ablation analysis assessing the contribution of Arpeggio-defined edges and interaction features using shuffle-groups-out cross-validation (11 splits). A significant interaction between edge definition and feature type was observed (two-way repeated measures ANOVA with Geisser-Greenhouse correction; F(1,10) = 28.75, *p* = 0.0003). Removing either Arpeggio-defined edges or interaction features significantly reduces performance (FDR-corrected multiple comparisons using the Benjamini, Krieger, and Yekutieli method; *q < 0.05). Error bars indicate mean ± SEM. (E) DeorphaNN (mAP = 0.45 ± 0.042) outperforms peptide pLDDT (mAP = 0.32 ± 0.039; same data as in Figure 1) in agonist prioritization (Wilcoxon matched-pairs signed-rank test; ***p < 0.001). Error bars indicate mean ± SEM. The *x* axis is plotted on a log10 scale. (F) Heatmap of AP values from (E), showing that DeorphaNN prioritizes agonists missed by peptide pLDDT.

To integrate features from different subregions, we generated graph representations of GPCR-peptide complexes (Figure 4A), enabling a GNN to capture complex relational data (e.g., residue interactions). The graph representations consist of two types of nodes corresponding to GPCR residues and peptide residues. Intermolecular edges between these nodes were defined using Arpeggio,^40^ which identifies biophysically meaningful interatomic interactions in protein structures based on spatial and physicochemical criteria (Figure 4B). Only GPCR residues predicted to interact with peptide residues via Arpeggio-identified interactions—specifically hydrogen bonds, ionic, halogen, aromatic, hydrophobic, carbonyl, polar, or metal-complex interactions—were included as nodes, ensuring the graphs focus on the predicted binding site. All peptide residues were included as nodes, since their smaller length permits their inclusion without excessive computational burden. To preserve intra-molecular connectivity of the peptides, sequential edges were added based on sequence adjacency.

GPCR and peptide nodes were loaded with embeddings derived by residue-specific pooling of the pair representation’s GPCR or peptide subregion, respectively (Figure 4C). Specifically, for each residue *i*, we averaged the *i*-th row and th*e i*-th column across all features of the respective subregion matrix, yielding a 128-dimensional embedding capturing residue-level structural and interaction information. Edges between GPCR and peptide nodes were loaded with pairwise interaction embeddings. To maintain consistent edge dimensions, edges between peptide nodes were assigned neutral embeddings set uniformly to the mean edge-attribute vector across all edges. This design ensures that node embeddings encode residue-specific information, incorporating both local and global context from the pair representation, while interaction edges encode intermolecular residue-residue interaction context.

We trained an attention-based GNN to assign an agonist-likelihood score for each GPCR-peptide graph. The model performs attention-based message passing over the constructed graphs and produces a graph-level score via global pooling. For each fold, the GPCRs listed in Table S1 were held out as the test set, grouped according to phylogenetic similarity (Data S1). Hyperparameter tuning was performed using GPCRs that were excluded from the test fold, ensuring no overlap with test data. The remaining GPCR clusters formed the training set, with negative data points subsampled. Model performance was evaluated per fold, and final predictions were obtained by aggregating outputs from an ensemble of bootstrap-trained models. All evaluation metrics reported in the figures were calculated using the held-out test sets, ensuring that performance reflects predictions on GPCRs not seen during training or hyperparameter tuning.

### Impact of graph structure and edge embeddings on GNN performance

To assess how graph structure and edge features influence performance, we conducted an ablation study (Figure 4D). First, we replaced Arpeggio-defined interaction edges with distance-based edges (residues *≤*6 Å apart), resulting in a reduction in AP when evaluated via shuffle-groups-out cross-validation. Next, we removed pairwise interaction embeddings from Arpeggio-defined edges, which decreased performance (Figure 4D). Interestingly, when both changes were combined—using distance-based edges and omitting edge features—the model slightly outperformed either single ablation alone. This combined ablation still achieved lower AP than the complete graph structure (Arpeggio edges with edge features).

Distance-based edges may introduce noise by capturing non-functional contacts lacking physicochemical specificity, while Arpeggio edges alone may be insufficient without the context provided by pairwise interaction embeddings. The complete graph, combining Arpeggio edges with edge features, outperformed all ablations, suggesting a synergistic effect of biophysically meaningful edges and interaction-aware features, although the observed differences are modest. The final model, called DeorphaNN, was trained on the *C. elegans* dataset using this framework and achieved an mAP of 0.45 (Figure 4E; Figures S4A and S4B). When evaluated on completely held-out peptide families, DeorphaNN achieved an average PR-AUC of 0.422 ± 0.080 across ten families tested (Table S2), though performance varied across families. Although there were only two class B GPCRs in the training dataset, both achieved an AP score > 0.50. AP scores were not correlated with either the multiple sequence alignment (MSA) normalized effective number of sequences (NEFF) of the GPCR, the GPCR’s pLDDT, or the maximum percent sequence identity of the GPCR TM region to others in the training set, suggesting that DeorphaNN’s ability to prioritize agonists does not depend on the availability of evolutionary information for the GPCR, AF2’s modeling confidence, or similarity to other GPCRs in the dataset (Figures S4C–S4G). Importantly, DeorphaNN can prioritize agonists that are overlooked by peptide pLDDT (Figure 4F), demonstrating its ability to identify agonists independently of pLDDT-based prioritization.

### Validation on phylogenetically diverse data and comparison to state-of-the-art

**Figure 5:**
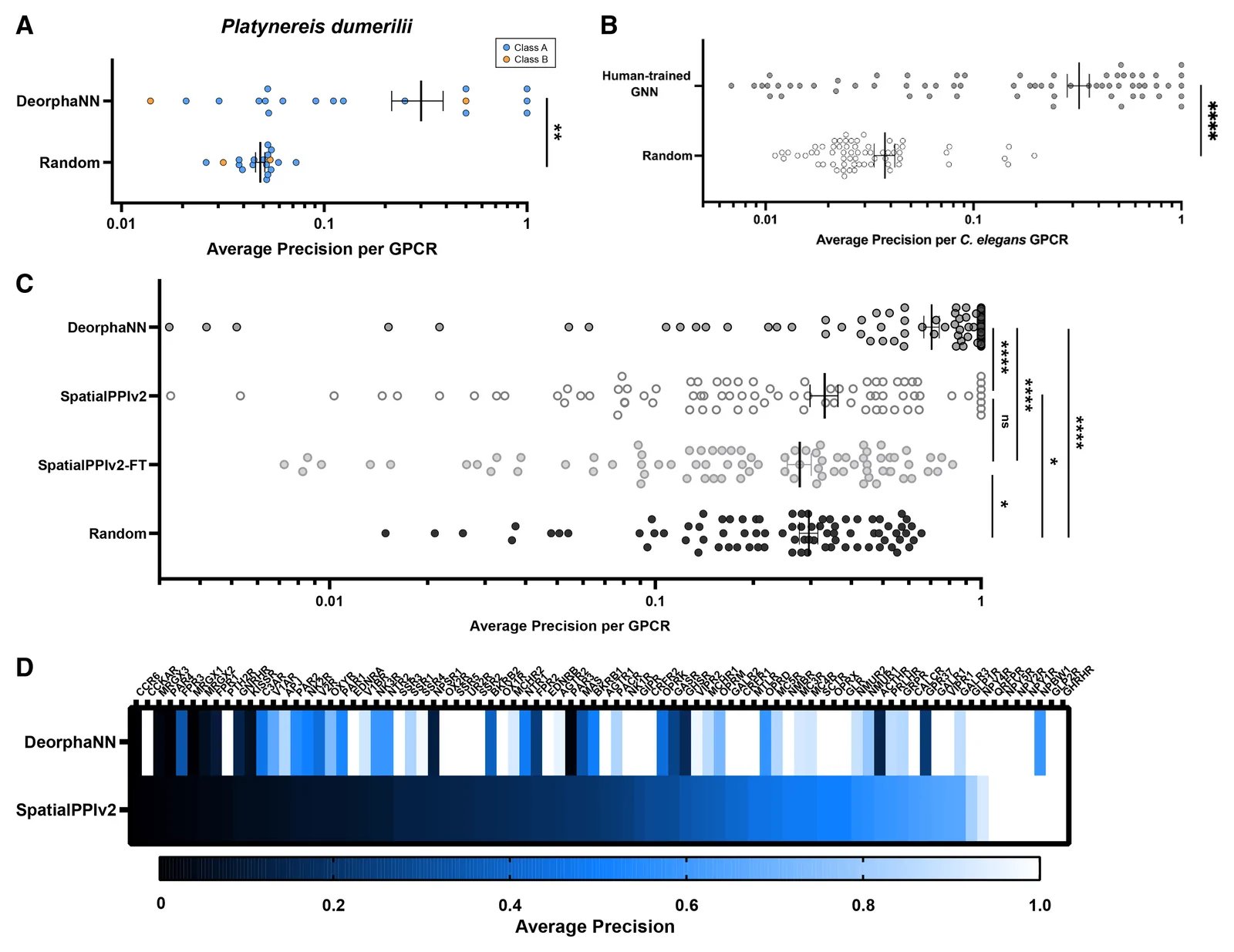
DeorphaNN generalizes to non-*C. elegans* datasets (A) DeorphaNN, trained on *C. elegans* data, prioritizes peptide agonists for GPCRs from *Platynereis dumerilii* (mAP = 0.30 ± 0.086), performing significantly better than random ranking (mAP = 0.05 ± 0.003) (Wilcoxon matched-pairs signed-rank test; **p < 0.01). Class A and B GPCRs are depicted in blue and orange, respectively. (B) DeorphaNN trained on a human GPCR-peptide agonist dataset augmented with ESM-2-guided decoy (putative non-agonist) pairs significantly outperforms random ranking when evaluated on the *C. elegans* dataset (mAP = 0.31 ± 0.044 vs. 0.035 ± 0.004; Wilcoxon matched-pairs signed-rank test; ****p < 0.0001). (C) DeorphaNN (trained on the *C. elegans* dataset) significantly outperforms SpatialPPIv2, SpatialPPIv2 fine-tuned on the *C. elegans* dataset, and random ranking in human GPCR-peptide agonist prioritization (mAP = 0.71 ± 0.038, 0.33 ± 0.033, 0.28 ± 0.023, and 0.30 ± 0.019, respectively). Statistical significance was determined using the Friedman test with Dunn’s correction (*p < 0.05, ****p < 0.0001). (D) Heatmap for the AP values from (C), illustrating that DeorphaNN prioritizes agonists overlooked by SpatialPPIv2. The *x* axis in (A)–(C) is plotted on a log10 scale. Error bars indicate mean ± SEM.

To assess whether DeorphaNN captures conserved mechanisms of GPCR-peptide agonism rather than species-specific patterns, we evaluated its performance on a dataset from the marine annelid, *Platynereis dumerilii*.^10^ This dataset contains 18 GPCRs and 122 peptides that were tested in a similar experimental pipeline as the *C. elegans* training dataset (CHO Gα_16_-mediated calcium signaling assay). The percent identity of the TM region of each GPCR to its nearest *C. elegans* training homolog ranged from 25.6% to 47.3% (median 33.8%; Figure S5A). We utilized DeorphaNN to rank *Platynereis* peptides for each GPCR based on predicted agonist potential. The mAP across all *Platynereis* GPCRs was significantly greater than the baseline expected with random ranking (Figure 5A). The distribution of AP values appears to show clusters of high- and low-performing receptors, but stratifying by GPCR class reveals no clear patterns, and AP was not correlated with GPCR TM sequence identity to the training set (Figures S5B and S5C). To investigate whether the model relies on peptide-likeness rather than receptor-interface determinants, we examined agonist similarity to training peptides. DeorphaNN AP showed no significant correlation with average maximum agonist cosine similarity of ESM-2 peptide embeddings to the training set and did not differ between high- and low-similarity receptor groups (Figures S5D–S5F).

To probe DeorphaNN’s generalization to human GPCR-peptide interactions, we utilized a literature-curated dataset of experimentally confirmed agonist interactions.^41^ The percent identity of the TM region of each human GPCR to the nearest training homolog ranged from 23.5% to 41.7% (median 31.2%; Figure S6A). While this dataset includes human GPCR-agonist pairs, it lacks non-agonist interactions. To address this gap, we generated synthetic non-agonist GPCR-peptide pairs using a dissimilarity-based approach inspired by principles from prior work,^41^ leveraging ESM-2^42^ language model embeddings to quantify peptide dissimilarity. By calculating the distance between each peptide’s ESM-2 representation and that of the cognate agonists, we selected peptides with a distance exceeding an empirical threshold chosen to ensure at least 3 non-agonists per GPCR. To validate the biological relevance of these synthetic pairs, we trained the GNN on the augmented human dataset and tested it on the *C. elegans* dataset, which contained no synthetic data (Figure 5B). This model achieved scores significantly higher than the random baseline, confirming that the augmented human dataset reliably captures real-world interaction patterns and supporting its utility for benchmarking.

We benchmarked DeorphaNN (trained on *C. elegans* data) on the augmented human dataset, achieving an mAP of 0.71—significantly higher than the random baseline of 0.30 (Figure 5C). The AP of each GPCR was not correlated with sequence identity to the nearest training homolog nor with agonist similarity to training peptides, indicating that DeorphaNN’s performance is not driven by similarity to the training set (Figures S6B–S6F).

To compare to the state-of-the-art, we evaluated SpatialPPIv2, a model designed for protein-protein interaction prediction that leverages large language models and graph attention networks.^43^ We assessed the pretrained model, as well as a model fine-tuned using the *C. elegans* training dataset (Figures S7A and S7B). On the same augmented human dataset, both the pretrained and fine-tuned SpatialPPIv2 models outperform random ranking but are not significantly different from one another. DeorphaNN significantly outperformed both versions of SpatialPPIv2. We also evaluated DeorphaNN’s performance on a human GPCR-peptide dataset that was recently used to benchmark AlphaFold3^44^ and found that DeorphaNN significantly outperforms AF3 in prioritizing peptide agonists for human GPCRs (Figures S7C and S7D). This highlights that our specialized GNN—which integrates structural predictions and interaction data—captures biologically relevant features specific to GPCR-peptide agonism more effectively than general protein-protein interaction models.

Notably, despite the sparsity of class B GPCRs in the training dataset, DeorphaNN achieved an AP > 0.95 for all twelve of the class B GPCRs in the human dataset (Figure S6G). To further interrogate this result, we generated a within-class decoy set restricted to agonists of class B GPCRs. For each receptor, peptides were ranked by distance in ESM-2 embedding space relative to the cognate agonists, and the five most dissimilar peptides were selected as decoys. These within-class decoy peptides were longer and more similar to cognate agonists in ESM-2 embedding space than the original class-agnostic decoys (Figures S6H and S6I). DeorphaNN nevertheless retained high discriminative performance, achieving a within-class mAP of 0.96 (Figure S6G; Data S3), suggesting that DeorphaNN’s performance on class BGPCRs is not an artifact of class-dependent ligand similarity.

### Discovery of novel peptide agonists for orphan GPCRs identified by virtual screening

Some peptide agonists were identified after the system-wide experimental screen of GPCR-peptide interactions in *C. elegans*. Indeed, peptidomics and comparative genomics studies have since expanded the number of peptides known in *C. elegans* beyond those included in the training dataset,^45–47^ providing an opportunity to reassess *C. elegans* GPCRs with a broader set of candidate ligands. The receptor NPR-34, an ortholog of the elevenin peptide receptor family with maximum sequence identity of 29.9% to GPCRs in the training set, was not activated by any of the peptides in the training dataset and was therefore not included in the training data. However, subsequent experiments identified the *C. elegans* elevenin peptide, SNET-1-1, as a cognate ligand of this receptor.^8^ This agonist ranks first out of 364 candidates (339 peptides from the training dataset plus 25 recently identified peptides that were not included in the training data [Data S1]) by DeorphaNN’s predictions (Table 1). Similarly, the GPCR SEB-2, an ortholog of the calcitonin receptor family with 22% maximum sequence identity to training set GPCRs, was not included in the dataset used to train DeorphaNN as it showed no activation in the original screen. The calcitonin-like peptide NLP-73-3 was tested in the original screen, but the synthesized peptide lacked a disulfide bridge.^8^ When SEB-2 was later challenged with a properly folded version of NLP-73-3, it showed robust activation.^8^ Indeed, NLP-73-3 is ranked first among all candidate peptides for SEB-2 by DeorphaNN (Table 1).

Additionally, we applied DeorphaNN to the human GPCR, GPR139, a class A orphan GPCR with emerging pharmacology.^48^ Dynorphin-derived peptides were recently identified as potential GPR139 agonists in a recent screen of human neuropeptides.^49^ Evaluation of this same neuropeptide library with DeorphaNN positions two dynorphin-derived peptides at the top of the ranked list (Data S4). We then performed a virtual screen of a reference library of >1,000 putative human peptides^14^ and supplemented this with dynorphin-derived peptides. Two dynorphin-derived peptides ranked in the top 2% of all peptides by DeorphaNN score (Data S4). This retrospective analysis supports DeorphaNN’s practical value for guiding experimental screening efforts.

**Figure 6:**
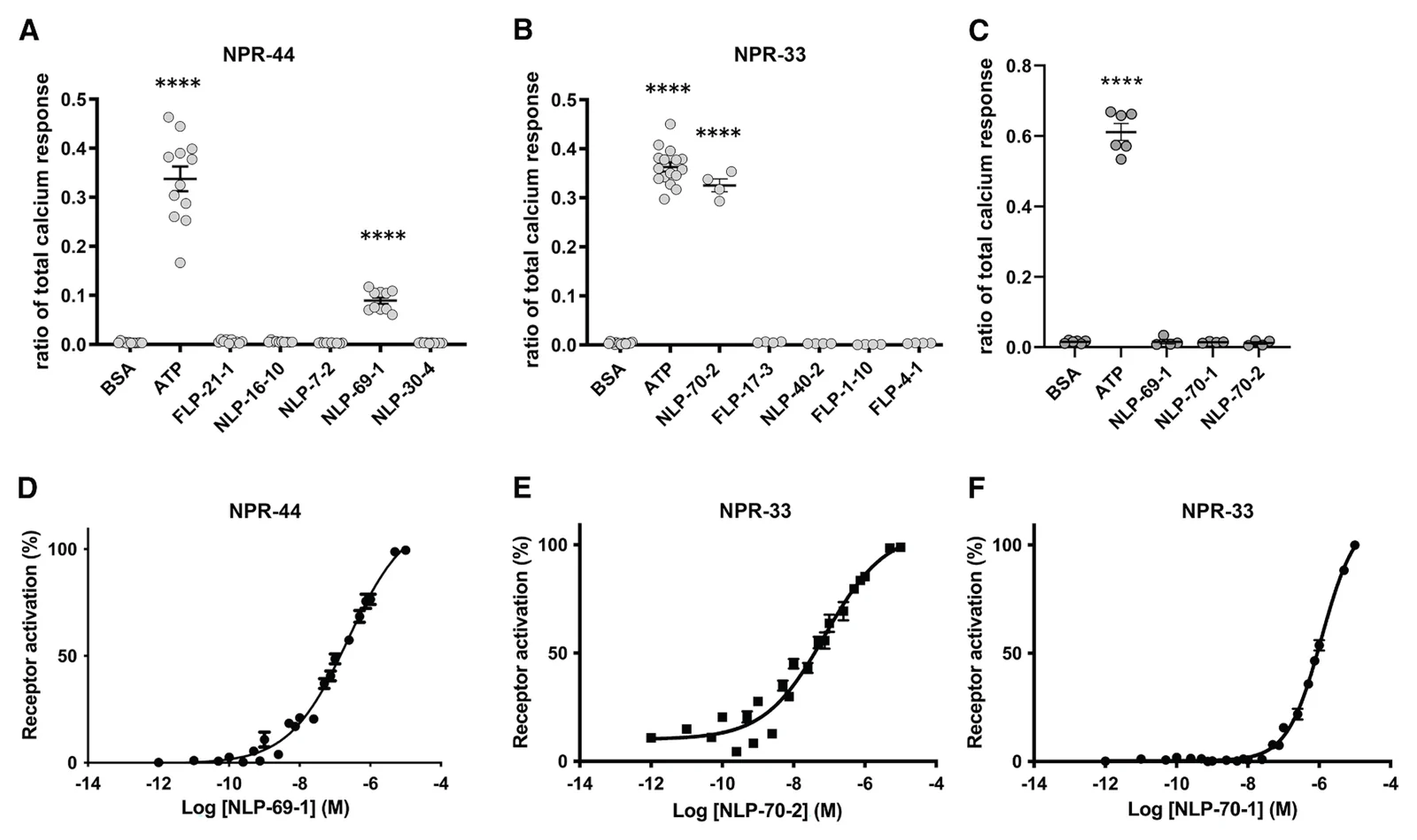
DeorphaNN-guided identification and experimental validation of agonists for *C. elegans* GPCRs (A–C) The top five DeorphaNN-ranked peptides for NPR-44 and NPR-33 were tested at 10 μM in CHO cells co-expressing Gα16 and aequorin, with ATP serving as a positive control. Statistical significance was assessed by one-way ANOVA with Dunnett’s multiple comparisons test relative to bovine serum albumin (BSA) vehicle; ****p = < 0.0001. Error bars indicate SEM. (A) NLP-69-1, ranked 4/364 for NPR-44, produced a significant increase in calcium response (*n ≥* 9). (B) NLP-70-2, ranked 1/364 for NPR-33, produced a significant increase in calcium response (*n ≥* 4). (C) Peptides do not affect calcium levels in CHO cells transfected with an empty (pcDNA3.1) vector in the absence of transfected GPCR (*n ≥* 4). (D–F) Concentration-response curves showing calcium mobilization in CHO cells co-expressing the indicated GPCR in response to peptide stimulation. *n* = 6 for all assays. (D) NPR-44 activation by NLP-69-1 (EC_50_ = 231.7 nM; 95% confidence interval [CI] 153.6–309.7 nM). (E) NPR-33 activation by NLP-70-2 (EC_50_ = 76.3 nM; 95% CI 50.3–128.0 nM). (F) NPR-33 was also activated by another *nlp-70* gene peptide, NLP-70-1 (ranked 167/364), at a lower potency (EC_50_ = 1.16 μM; 95% CI 1.00–1.38 μM).

Finally, we applied DeorphaNN to two orphan *C. elegans* GPCRs, NPR-44 and NPR-33. Both receptors demonstrated <31% maximum percent sequence identity of the TM region to GPCRs in the training dataset (Table 1), and neither was used to train DeorphaNN. We experimentally tested the five highest-ranked candidate agonists in our Gα_16_-based receptor activation assay in CHO cells (Figures 6A–6C). Of the five peptides tested per GPCR, one peptide activated NPR-44, and one activated NPR-33, demonstrating that DeorphaNN’s rankings can identify agonists within the top 5 of 364 candidates to reduce the need for exhaustive screening. For NPR-44, we observed robust activation by NLP-69-1 (EC_50_ value of 231.7 nM), ranked 4^th^ out of 364 peptides by DeorphaNN (Table 1; Figure 6D; Data S4). In addition, NPR-33 was potently activated by NLP-70-2 (EC_50_ value of 76.3 nM), the highest-ranked peptide (Table 1; Figure 6E; Data S4). No response was seen with any of the peptides in cells transfected with an empty vector control (Figure 6C). Given that GPCRs are often activated by multiple peptides within the same peptide-encoding gene (Figure S1A), we also tested NLP-70-1 (ranked 167/364 by DeorphaNN for NPR-33), another putative peptide encoded by the *nlp-70* gene. Although we detected receptor activation, NLP-70-1’s EC_50_ value (1.16 μM; Figure 6F) is considerably higher than that of NLP-70-2. This suggests DeorphaNN may prioritize agonists with specific interaction patterns that correlate with higher potency. Indeed, the majority of agonist interactions in the *C. elegans* dataset exhibit high potency (EC_50_ < 500 nM),^8^ which may explain why lower-potency agonists like NLP-70-1 were ranked lower by DeorphaNN.

## Discussion

In this study, we present DeorphaNN, a GNN-based pipeline for prioritizing GPCR-peptide agonist interactions *in silico* as a strategy for GPCR deorphanization. Previous studies have shown that pLDDT metrics from AlphaFold predictions correlate with interaction affinity,^30,31,50^ consistent with our findings that pLDDT partially discriminates agonists from non-agonists. However, affinity alone is insufficient to determine agonist function,^34,51^ as it does not capture additional mechanisms required for functional activation, such as intrinsic efficacy^52^—the propensity of a ligand to induce conformational changes in the GPCR that activate downstream G protein signaling.

Agonist binding induces conformational changes in the GPCR that drive conserved TM rearrangements.^37,53,54^ A key feature of this process is the outward movement of TM6,^55^ facilitated by kinking at conserved motifs and disruption of ionic interactions between TM3 and TM6/7.^56–58^ These rearrangements reconfigure the surface of the orthosteric binding pocket,^59,60^ enhancing affinity for agonists.^61^ DeorphaNN capitalizes on these mechanisms by incorporating active-state structural templates, enabling the modeling of GPCR-peptide complexes in their active conformations to prioritize peptides that both bind and stabilize the active-state binding pocket.

Agonists activate GPCRs through interactions with key ‘‘toggle switch’’ residues in the binding pocket.^62,63^ This activation is thought to require two distinct types of intermolecular interactions: ‘‘anchor’’ interactions that provide stability for ligand binding and ‘‘driver’’ interactions that directly engage with toggle switches to induce GPCR conformational changes.^64–66^ To enable pattern recognition of these interactions, we engineered DeorphaNN to utilize Arpeggio-defined edges—capturing interatomic interactions^40^—and AF pairwise embeddings—encoding implicit biophysical relationships between residues.^26^ By contrast, SpatialPPIv2—a state-of-the-art model for predicting general protein-protein interactions^43^—lacks this context, explaining its reduced performance in differentiating agonist from non-agonist interactions compared with DeorphaNN, even when fine-tuned on DeorphaNN’s training dataset.

Despite being trained on a relatively small number of GPCR-agonist pairs from *C. elegans*, its performance in retrospective benchmarks supports DeorphaNN’s computational generalization across species. This result reflects the effective application of inductive transfer learning principles, which enable models pretrained on large datasets to be fine-tuned for specialized tasks with limited data.^67,68^ In our work, DeorphaNN leverages frozen embeddings acquired without changing the weights of the AF2 algorithm, which was pretrained on approximately 170,000 protein structures from diverse species,^25^ and adapts it for the specialized task of GPCR-peptide agonism prediction. The model’s ability to prioritize agonist interactions in phylogenetically distinct species such as *Platynereis dumerilii* and humans suggests that the implicit biophysical principles learned by AF2 were transferred to this specialized task.

Compared with existing methods, DeorphaNN represents a significant advancement in GPCR-peptide agonist identification. Peptide descriptor (PD)-incorporated support vector machine (SVM) predicts peptide agonists for GPCRs using an SVM trained on PDs that represent sequence patterns of 1–5 residues encoded into numeric arrays.^41^ DeorphaNN, on the other hand, leverages structural predictions, enabling it to learn from three-dimensional relationships and biophysical patterns not captured by primary sequence alone. GPCRVS employs deep neural networks, gradient boosting machines, and AutoDock Vina to predict compound activity, selectivity, and binding affinity for small molecules and peptides but is restricted to GPCRs within its training data and can only effectively screen N-terminal fragments of peptides (six residues or smaller).^69^ By contrast, DeorphaNN lacks these limitations.

In this study, we use DeorphaNN to accelerate the identification of agonists for two previously orphan GPCRs in *C. elegans*. Beyond facilitating the discovery of novel signaling pathways, DeorphaNN may aid in accelerating drug discovery efforts, as recent advances in machine learning-based synthetic peptide design have produced tools for generating synthetic ligands for GPCRs.^70,71^ DeorphaNN has the potential to complement these tools by prioritizing candidate peptide agonists for experimental validation and reducing the need for exhaustive screening. Following deorphanization, the identification of peptide agonists is also relevant to structural studies of GPCR-ligand complexes and agonist-induced conformational changes,^72–74^ as high-affinity agonists help stabilize the receptor in a defined active state.^75^ By prioritizing putative peptide agonists, DeorphaNN’s potential to aid in deorphanization efforts and agonist discovery lays a foundation for both structural and mechanistic studies of GPCR-peptide interactions.

### Limitations of the study

Despite DeorphaNN’s ability to generalize beyond receptors in the training dataset, its performance varied among GPCR families. Structural and sequence-quality metrics did not correlate with model performance, nor did similarity to receptors in the training set, making it difficult to estimate reliability for novel receptors *a priori*. Determining which factors affect DeorphaNN’s predictive performance is an important area for future investigation to allow the development of a heuristic indicator of the model’s reliability for a given GPCR.

The training dataset itself may contribute to this variability. We used data derived from a single Gα_16_-mediated receptor activation screen, which introduces both pathway bias and the possibility of false negatives. As with most protein-protein interaction studies, reliable negative data are difficult to obtain: the absence of activity in a particular assay does not guarantee a true biological negative.^8,14^ While the promiscuous Gα_16_ subunit enables many GPCRs to signal through the PLCβ pathway, the effectiveness of this signaling can be ligand specific,^76^ and peptides that preferentially or exclusively engage other pathways (such as through Gs-mediated cAMP or Gq-mediated IP_1_) may be miscategorized as non-agonists in the dataset. This Gα_16_-bias may present a hard ceiling on recall of Gs/β-arrestin-biased agonists. Some peptides may also be misclassified as non-agonists for highly promiscuous receptors, as not all peptides from the same precursor gene were tested against these receptors for agonist-induced activity.^8^

DeorphaNN relies on AF2 to accurately model GPCR-peptide complexes. AF2 predictions lack an explicit membrane context, and AF2-predicted GPCR structures show deviations in ligand-binding pockets and extracellular domains compared with experimentally determined structures.^77,78^ Another limitation is the inability of AF2 to model post-translational modifications. Post-translational modifications frequently influence GPCR and peptide structure, affecting ligand binding and receptor activation. For GPCRs, extracellular post-translational modifications such as tyrosine sulfation, glycosylation, and proteolytic cleavage may alter ligand binding.^79^ For peptides, post-translational modifications such as C-terminal amidation or pyroglutamation are often important for agonist activity.^34^ Indeed, amidation has been shown to influence biological activity,^80^ and the omission of such modifications in AF2 predictions may result in lost interaction information. Emerging structure prediction models capable of explicitly representing post-translational modifications^81–83^ may provide an opportunity to evaluate the impact of these features on DeorphaNN’s predictive performance.

Finally, DeorphaNN’s generalizability to human GPCRs is supported primarily by retrospective benchmarks drawn from published interactions. These datasets are likely enriched for well-studied receptor families, and consequently, retrospective benchmarks may overestimate performance on previously uncharacterized GPCR-peptide pairs. Future studies that use DeorphaNN to prioritize candidates for experimental validation will provide a more rigorous assessment of its utility for deorphanization and agonist discovery.

## Resource Availability

### Lead contact

Requests for further information and resources should be directed to and will be fulfilled by the lead contact, Larissa Ferguson (LariFerg@gmail.com).

### Materials availability

The CHO-K1 cell line stably expressing mitochondrial-targeted apo-aequorin is under MTA and cannot be freely distributed.

### Data and code availability

All datasets utilized in this study are available as supplemental materials and in the associated Zenodo repository (DOI: 10.5281/zenodo.20862123). The DeorphaNN code is available at https://github.com/Zebreu/DeorphaNN (DOI: 10.5281/zenodo.20865768). Any additional information required to reanalyze the data reported in this paper is available from the lead contact upon request.

## Star★Methods

Detailed methods are provided in the online version of this paper and include the following:

- KEY RESOURCES TABLE
- EXPERIMENTAL MODEL AND STUDY PARTICIPANT DETAILS
  - Cell lines
- METHOD DETAILS
  - Datasets
  - Phylogenetic analysis of GPCRs
  - AlphaFold2
  - Mean Average Precision (mAP)
  - Supervised learning algorithms
  - Arpeggio
  - Graph construction
  - Model architecture and training
  - MSA diversity
  - Peptide similarity analysis
  - AlphaFold3 Benchmark Comparison
  - Aequorin-based GPCR activation assay
- QUANTIFICATION AND STATISTICAL ANALYSIS

## Star★Methods

### Key Resources Table

**Table:** 

REAGENT or RESOURCE	SOURCE	IDENTIFIER
Chemicals, peptides, and recombinant proteins		
Zeocin selection reagent	Invitrogen	Cat#R25001
1% Penicilin/Streptomycin Mixture	Gibco	Cat#15140-122
Fetal Bovine Serum	Sigma-Aldrich	Cat#F7524
Lipofectamine LTX, Plus Reagent	Invitrogen	Cat#15338-500, Cat#11514015
Deposited data		
AF2 outputs (relaxed pdbs and pair representations) for *C. elegans* dataset	This study	10.5281/zenodo.20862123
DeorphaNN	This study	https://github.com/Zebreu/DeorphaNN10.5281/zenodo.20865768
DeorphaNN scores, human orphan GPCRs	This study	10.5281/zenodo.20862123
Experimental models: Cell lines		
CHO/mtAEQ/Gα_16_	PerkinElmer	ES-000-A24
Software and algorithms		
Prism	Graphpad	https://www.graphpad.com/scientific-software/prism/
ScreenWorks System control software	Molecular Devices	FLIPR Tetra control program
Arpeggio	Jubb et al.^45^	https://biosig.lab.uq.edu.au/arpeggioweb/
ColabFold v1.5.5	Mirdita et al.^84^	https://github.com/sokrypton/ColabFold
DeepTMHMM	Hallgren and Winther^36^	https://dtu.biolib.com/DeepTMHMM
NEFFy	Haghini et al.^85^	https://github.com/Maryam-Haghani/NEFFy
ESM-2	Lin et al.^46^	https://github.com/facebookresearch/esm
pDockQ	Bryant et al.^37^	https://gitlab.com/ElofssonLab/FoldDock/
AF-Multistate	Xue et al.^34^	https://github.com/huhlim/alphafold-multistate
AlphaFold Multimer	Evans et al.^31^	https://github.com/google-deepmind/alphafold

### Experimental Model and Study Participant Details

#### Cell lines

Peptide agonist activity was tested in CHO-K1 cells stably expressing mitochondrial-targeted apo-aequorin and a promiscuous human Gα_16_ protein (CHO/mtAEQ/Gα_16_, ES-000-A24, PerkinElmer). Cells were cultured in Dulbecco’s modified Eagle’s medium (DMEM)/Nutrient Mixture F-12 Ham (Sigma-Aldrich), supplemented with 10% fetal bovine serum (FBS, Sigma-Aldrich), 1% penicillin/streptomycin mixture (10.000 units/ml penicillin and 10 mg/mL streptomycin, Gibco), and 250 μg/mL zeocin. Cells were grown in 37 ^*°*^C, 5% CO_2_ and high relative humidity.

### Method Details

#### Datasets

The *C. elegans* training dataset was derived from a previously published system-wide screen using a CHO Gα_16_-mediated calcium signalling assay to test peptide agonist activity for over 160 predicted peptide-activated GPCRs in *C. elegans*.^8^ To accommodate AF-multimer’s limitations regarding post-translational modifications, we preprocessed peptide sequences by removing C-terminal glycine residues for amidated peptides and N-terminal glutamine residues for pyroglutamated peptides. Disulfide bridges were not changed. After removing duplicate sequences and peptides shorter than 3 residues, 339 unique peptides remained. GPCRs that lacked any concentration-dependent agonist responses in the screen were excluded, reducing the likelihood that an observed lack of agonist activity was the result of experimental limitations. Additionally, we excluded the GPCR DMSR-8-1, which has the same agonist interaction profile as the other DMSR-8 isoform (DMSR-8-2) and only differs in sequence by an addition of four N-terminal residues. This filtering process resulted in a final training dataset comprising 65 unique GPCRs derived from 55 genes. The full list of GPCRs and peptides included in the *C. elegans* training dataset is provided in Data S1.

The *Platynereis dumerilii* dataset used for cross-species validation was derived from a previously published report.^10^ We processed all peptides with modified residues as above and omitted peptides with D-amino acids, resulting in 122 peptides. We similarly omitted GPCRs that had no confirmed agonists among the included peptides, resulting in 18 receptors and 23 agonist interactions.

For the human dataset, we utilized a previously published list of GPCR–peptide agonist interactions compiled across diverse species, including vertebrates and invertebrates.^41^ We limited our analysis to agonist interactions involving human GPCRs, while using peptides from all species (excluding those longer than 50 residues) to augment the dataset with synthetic non-agonist interactions. Specifically, we generated ESM2-150M^42^ embeddings for each peptide and identified, for each GPCR, peptides with Euclidean distances > 3.9 from known agonists, ensuring at least 3 putative non-agonists per GPCR. This process yielded an augmented dataset containing 345 agonist and 3370 non-agonist GPCR-peptide pairs across 82 human GPCRs. For within-class decoys, 5 peptides with the largest mean distances were selected as decoys from a list of peptides that are cognate agonists for Class B GPCRs.

In all datasets, we omitted GPCRs that were not predicted by DeepTMHMM to have the characteristic GPCR structure (seven transmembrane domains, with an extracellular N-terminal and an intracellular C-terminal). The full list of GPCRs and peptides included in the validation datasets (*Platynereis* and human) are provided in Data S2.

The putative peptides evaluated with GPR139 are peptide cleavage variants previously identified as candidate ligands.^14^ We limited the library to peptides between 3–37 amino acids in length. The neuropeptides evaluated with GPR139 were those from a previously published experimental screen,^49^ omitting duplicate peptides and those with non-canonical amino acids. All peptide sequences are provided in Data S4.

#### Phylogenetic analysis of GPCRs

For comparisons among GPCRs within the *C. elegans* training dataset, amino acid sequences were aligned using MAFFT with the L-INS-i algorithm and default parameters (BLOSUM62 scoring matrix, gap-opening penalty 1.53, offset value 0.123).^86^ To focus on transmembrane regions, aligned sequences were trimmed with ClipKIT using the gappy mode with a gap threshold of 0.9.^87^ Phylogenetic trees were inferred using maximum-likelihood methods with IQ-TREE 2 under the LG+G+I substitution model with 1,000 ultrafast bootstrap replicates.^88^ GPCRs separated by <1.48 expected substitutions per site were clustered using Geneious Prime. Trees were visualized using the Interactive Tree of Life (iTOL) web server.^89^

For comparisons between GPCRs in the training dataset and GPCRs from other species, non-transmembrane regions were masked using DeepTMHMM.^90^ Masked sequences were then aligned using MAFFT with the L-INS-i algorithm and default settings,^86^ and pairwise percent identity was calculated using Geneious Prime.

#### AlphaFold2

Primary amino acid sequences for each GPCR-peptide complex were input together (GPCR:peptide) into AF-multimer^25,30^ using local ColabFold v1.5.5^91^ on a high-performance computing cluster. All modelling settings were left at default (model_type=alpha-fold2_multimer_v3, num_recycles=20, recycle_early_stop_tolerance=0.5, pairing_strategy=greedy, max_msa=auto), resulting in the generation of 5 predicted structures and corresponding confidence metrics. Hidden layer protein representations (single and pair) were collected for active state biased models only.

For confidence metric analyses, the model used for each GPCR–peptide pair was selected as the one with the highest ipTM, highest mean peptide pLDDT (defined as the average per-residue pLDDT across peptide residues), or lowest pocket PAE (defined as the mean pairwise predicted aligned error between peptide residues and GPCR residues within ±5 positions of the extracellular–membrane boundary as identified by DeepTMHMM), depending on the metric being analysed. To determine whether the peptide was modelled within the binding pocket, we carried out analysis on the predicted structure with the highest peptide pLDDT, measuring the minimum distance of peptide to binding pocket residues after averaging the location of all atoms in each residue and classifying peptides using a 12.5 Å cutoff.

For all protein representation analyses, representations across all five AF-multimer models were averaged to prevent algorithms from learning patterns specific to the different AF-multimer models.

To bias AF-multimer predictions towards GPCR active-state conformations, we first generated active-state GPCR templates using AF2-Multistate.^33^ This approach leverages state-annotated GPCR databases to guide predictions with a restricted MSA protocol. The top ranked active-state structures were processed by trimming intracellular residues with pLDDT values below 70 and all extracellular residues, based on DeepTMHMM^90^ annotations. These processed structures served as GPCR-specific templates in subsequent AF-multimer predictions for each GPCR–peptide pair, generating state-biased predictions. MSA reduction was not necessary as the templates were derived from the same sequence as the target GPCRs,^92^ and retaining the full MSA preserves coevolutionary signals that are vital for driving accurate inter-protein contact predictions.^93^

#### Mean Average Precision (mAP)

Ranking performance was quantified according to recommendation system metrics,^84^ using average precision (AP) computed across all peptides (*N*) per GPCR or GPCR gene. For each receptor, AP aggregates precision at every rank containing an agonist:

$$AP=\frac{\sum_{k}^{N}\left(\frac{\;\text{agonists}\;\text{in}\;\text{top}\;k}{k}\right)\times \;rel\;@k}{\;total\;agonists}$$

where *rel*@*k* equals 1 if the *k*-th ranked peptide is relevant (i.e. an agonist) and 0 otherwise. Mean average precision (mAP) averages AP equally across all GPCRs.

To establish a random baseline, we randomized peptide rankings 100 times per GPCR or GPCR family and computed the AP. The average of the 100 APs was then used as the random baseline AP.

#### Supervised learning algorithms

We implemented random forest classifiers using scikit-learn^94^ with default hyperparameters, leveraging their ability to perform implicit feature selection and assess feature importance. To maintain consistency across all cross-validation splits, we used identical random seed and data partitions. For Figures 3B and 3C, we employed stratified group 10-fold cross-validation. Grouping by GPCR was performed to minimize leakage of receptor-specific information between folds. Isoforms from the same GPCR gene were treated as a single unit, ensuring they appear together (55 unique GPCR genes), and class distribution was balanced by stratification. For Figures 3D–3F, we employed leave-one-group-out cross-validation. Reported APs for Figures 3E and 3F are an average of 5 different random seeds. Pooled scores are the average of the output scores from models trained independently on distinct representation types (peptide, bp:peptide, binding pocket). To generate a shuffled control, scores from two of the three models (peptide and binding pocket) were permuted across datapoints before averaging. To assess peptide-family generalization, we performed a held-out peptide-family analysis on the ten largest peptide families (by number of agonist interactions) to ensure sufficient true positives for a meaningful evaluation. As per-GPCR AP requires predictions across the full peptide candidate set, ROC-AUC and PR-AUC were reported (Table S2).

#### Arpeggio

The top-ranked GPCR-peptide structure according to peptide pLDDT was examined. Peptides that exhibited a minimum distance greater than 12.5 Å from the binding pocket were excluded from further analysis and added to the bottom of all rankings, as well as peptides with no GPCR contacts identified by Arpeggio. The position of each residue is defined by averaging the position of each residue atom. Structures were relaxed by Amber^95^ and analysed with Arpeggio^40^ to identify interatomic interactions between the GPCR and peptide. Specifically, we identified hydrophobic interactions, polar bonds, weak polar bonds, hydrogen bonds, weak hydrogen bonds, ionic bonds, aromatic ring interactions, van der Waals forces, and amide group interactions.

#### Graph construction

Graphs were constructed from GPCR–peptide interaction data obtained from AlphaFold2 structural predictions and pair representations. Each graph represents a GPCR–peptide complex, with nodes corresponding to individual residues. All peptide residues were included as nodes, while GPCR residues were included if they directly interacted with peptide residues or were within one degree of such interactions. Nodes were connected by two types of edges: intramolecular edges connecting nodes according to sequence adjacency, and intermolecular edges connecting GPCR and peptide nodes according to Arpeggio-identified interactions. For Figure 4D, spatial proximity-based edges (< 6 Å) were also utilized, computed by defining the residue’s position as the average position of the atoms that make up the residue.

Node features were derived using pair representations averaged over 5 models for each residue position. First, we processed the tensor ***P*** *∈* ℝ^*n*×*n*×128^—where *n* is the total residue count in the GPCR-peptide complex—to extract three sub-tensors:

$$\begin{array}{c} P^{\text{GPCR}}\in {\mathbb{R}}^{n_{GPCR}\times n_{GPCR}\times 128},\\ \;P^{\text{pep}}\in {\mathbb{R}}^{n_{pep}\times n_{pep}\times 128},\\ P^{\text{int}}=\frac{1}{2}\left(\left(P^{\text{GPCR},\text{pep}}\in {\mathbb{R}}^{n_{GPCR}\times n_{pep}\times 128}\right)+{\left(P^{\text{pep},\text{GPCR}}\in {\mathbb{R}}^{n_{pep}\times n_{GPCR}\times 128}\right)}^{T}\right), \end{array}$$

where *n*_*GPCR*_ and *n*_*pep*_ are residues of the GPCR and peptide, respectively, and T denotes transposition of the first two tensor dimensions.

We derived per-residue embeddings for GPCR and peptide nodes by applying cross-axis pooling independently to sub-tensors, ***P***^GPCR^ and ***P***^pep^. For each residue *i* in the sub-tensor *X ∈* {GPCR;pep}, we computed its embedding by averaging within each of the 128 features across the *i*-th row and the *i*-th column while correcting for (*i, i*) duplication:

$$z_{i}^{X}=\frac{\left(\sum_{k=1}^{n_{X}}P_{k,i,:}^{X}+\sum_{k=1}^{n_{X}}P_{i,k,:}^{X}\right)-P_{i,i,:}^{X}}{2n_{X}-1}.$$

Intermolecular edge features were derived from the interaction region of the pair representations (***P***^int^), with each intermolecular edge loaded with a 128-dimensional feature vector representing the interaction between the residue pairs connected by that edge. Intramolecular edges were assigned a uniform feature vector, computed as the mean-pooled representation of all intermolecular edge features across the graph.

#### Model architecture and training

The GNN architecture is based on the Graph Attention Network (GAT) framework, specifically utilizing the GATv2Conv^96^ layer implementation from PyTorch Geometric. The network begins with a Batch Normalization layer to standardize input features, ensuring stable training dynamics. This is followed by a GATv2Conv layer with 10 attention heads, a dropout rate of 0.5, and 64 hidden channels. The attention mechanism allows the model to dynamically weigh the importance of neighbouring nodes differently when aggregating information, enabling it to focus on relevant structural and interaction patterns within the graph. Random seeds were fixed for reproducibility ensuring consistent model initialization and dropout behavior, as well as controlling shuffling of training and negative examples.

After the convolutional layer, a rectified linear unit (ReLU)^97^ activation function introduces non-linearity to the model. For graph-level representation learning, a global mean pooling operation aggregates node embeddings across the entire graph, producing a fixed-size vector representation regardless of graph size. This pooled representation is then passed through a final linear layer with a dropout rate of 0.5 for regularization before being fed into a softmax function for classification. The model is trained using the AdamW optimizer^98^ with a learning rate of 0.0005 and employs CrossEntropyLoss^99^ with label smoothing to mitigate overfitting and improve generalization. During training, graphs are batched and processed using PyTorch Geometric’s DataLoader, with edge features incorporated where applicable.

For each validation fold, the training set was subsampled to maintain a balance between positive and negative examples. Specifically, for each positive example (agonist GPCR–peptide complex), four negative examples (non-agonist GPCR–peptide complex) were randomly selected, creating a balanced training set with a 1:4 ratio of positive to negative samples. The model selection was based on validation performance using group shuffle-split cross-validation, with early stopping to prevent overfitting. In this approach, the dataset was divided into groups based on GPCR families, and multiple random splits were generated where each split maintained a specified proportion of groups in the training and validation sets. This method ensures that the model is evaluated on previously unseen GPCR families while maintaining biological relevance in the validation process. The final model for each fold was chosen based on the highest validation average precision score achieved during training, and Optuna’s hyperparameter search used a fixed random seed for reproducibility of trial selection. Training was performed for 30 epochs, with the model evaluated periodically on the validation set to monitor convergence and generalization performance.

The final predictions reported were generated using an ensemble of 10 GNNs trained via bootstrap aggregating, with each model trained on a distinct 90% bootstrap sample of the training data. The ensemble outputs were aggregated to produce the final prediction scores.

#### MSA diversity

The sequence diversity of each GPCR was quantified using the Normalized Effective Number of Sequences (NEFF) computed from the corresponding MSA using NEFFy.^85^ NEFF values were obtained for the full GPCR sequence or averaged across the TM region (identified by DeepTMHMM^90^) using per-residue NEFF values.

#### Peptide similarity analysis

Peptide sequences were tokenized using the ESM-2 batch converter and passed through the pretrained ESM-2 model (esm2_t33_650M_UR50D) to generate per-residue embeddings. Per-sequence embeddings were obtained by averaging the perresidue embeddings along the residue dimension. Pairwise relationships between embeddings were quantified using cosine similarity. For analyses comparing agonists to the training set, cosine similarity was computed between each agonist peptide and all peptides in the training set, and the maximum similarity score was retained. Peptide similarity scores were obtained by averaging these maximum similarity values across the agonists of each GPCR. Dimensionality reduction was performed using UMAP to project high-dimensional sequence embeddings into a lower-dimensional space for visualization and clustering.^100^ Peptide similarity clusters were identified by applying agglomerative clustering to the pairwise Euclidean distances between UMAP embeddings.

#### AlphaFold3 Benchmark Comparison

We benchmarked DeorphaNN against AlphaFold3 (AF3) using a previously published human GPCR–peptide dataset,^44^ which was used to benchmark AF3’s performance in ranking peptide agonists for human GPCRs. After applying a 50 AA peptide cutoff, the dataset contained 82 GPCRs and 64 peptides, with one agonist and between 4–10 synthetic non-agonists per GPCR. AF3 interaction scores for each GPCR–peptide pair, calculated from the iPTM, were as reported in the original publication. DeorphaNN scores are provided in Data S3.

#### Aequorin-based GPCR activation assay

An aequorin-based calcium mobilization assay was used to measure GPCR activation following peptide application, as previously described.^8^ In brief, reverse pharmacology screening was performed using CHO-K1 cells expressing mitochondrial-targeted apoaequorin and promiscuous human Gα_16_. CHO cells were transfected with pcDNA3.1 plasmids containing the GPCR of interest and harvested two days after transfection. BSA served as a negative control for peptide-evoked calcium responses; ATP activates an endogenous receptor in CHO cells and was used as a positive control. Maximum calcium responses for normalization were obtained by lysing cells at the end of the assay.

#### Quantification and Statistical Analysis

Statistical analysis was performed in GraphPad PRISM version 10.2.2 for macOS and/or version 9.0.0 for Windows (GraphPad Software, San Diego, CA). Comparisons in each figure address distinct predefined hypotheses, and corrections for multiple comparisons were applied within each figure only. F1 and MCC were calculated using the threshold that maximized F1. All statistical tests were two-tailed.

## Supplementary Material

Supp Data 1

Supp Data 2

Supp Data 3

Supp Data 4

Supp Info

## Figures and Tables

**Table 1:** GPCR-peptide agonist pairs identified by DeorphaNN

GPCR	GPCR UniProt ID	Max GPCR TM% identity	Peptide	Peptide sequence	Rank by DeorphaNN	Source
NPR-34	G5EDR6	29.94	SNET-1-1	LDCRKFSFAPACRGIML	1/364	Beets et al.^8^
SEB-2	P30650	21.95	NLP-73-3	NRQCLLNAGLSQGCDF SDLLHAQTQARKFMSFAGP	1/364	Beets et al.^8^
NPR-44	Q9N5L5	26.25	NLP-69-1	LHRIGGNIVM	4/364	this paper
NPR-33	O62189	30.30	NLP-70-2	WYDWQNVPHALQQ	1/364	this paper
NPR-33	O62189	30.30	NLP-70-1	WYEWNNDMEIT	167/364	this paper

## Highlights

- AlphaFold has modest capability for prioritizing peptide agonists for GPCRs
- Active-state receptor conformations enhance agonist discrimination
- Pair representation subregions contain complementary predictive signals
- Integration into a graph neural network ‘‘DeorphaNN’’ accelerates agonist discovery
