# Supplementary material for: DeorphaNN: Virtual screening of GPCR peptide agonists using AlphaFold-predicted active-state complexes and deep learning embeddings
Source: Mol Cell. Author manuscript; Available in PMC 2026 Sep 8. (PMC7619462; doi:10.1016/j.molcel.2026.07.006)
Supplement: Supplementary file 1 [file EMS218177-supplement-Supp_Info.pdf]

Molecular Cell, Volume *86*

Supplemental information

DeorphaNN: Virtual screening of GPCR peptide agonists using AlphaFold-predicted active-state complexes and deep learning embeddings

Larissa Ferguson, Sébastien Ouellet, Elke Vandewyer, Christopher Wang, Zaw Wunna, Tony K.Y. Lim, William R. Schafer, and Isabel Beets

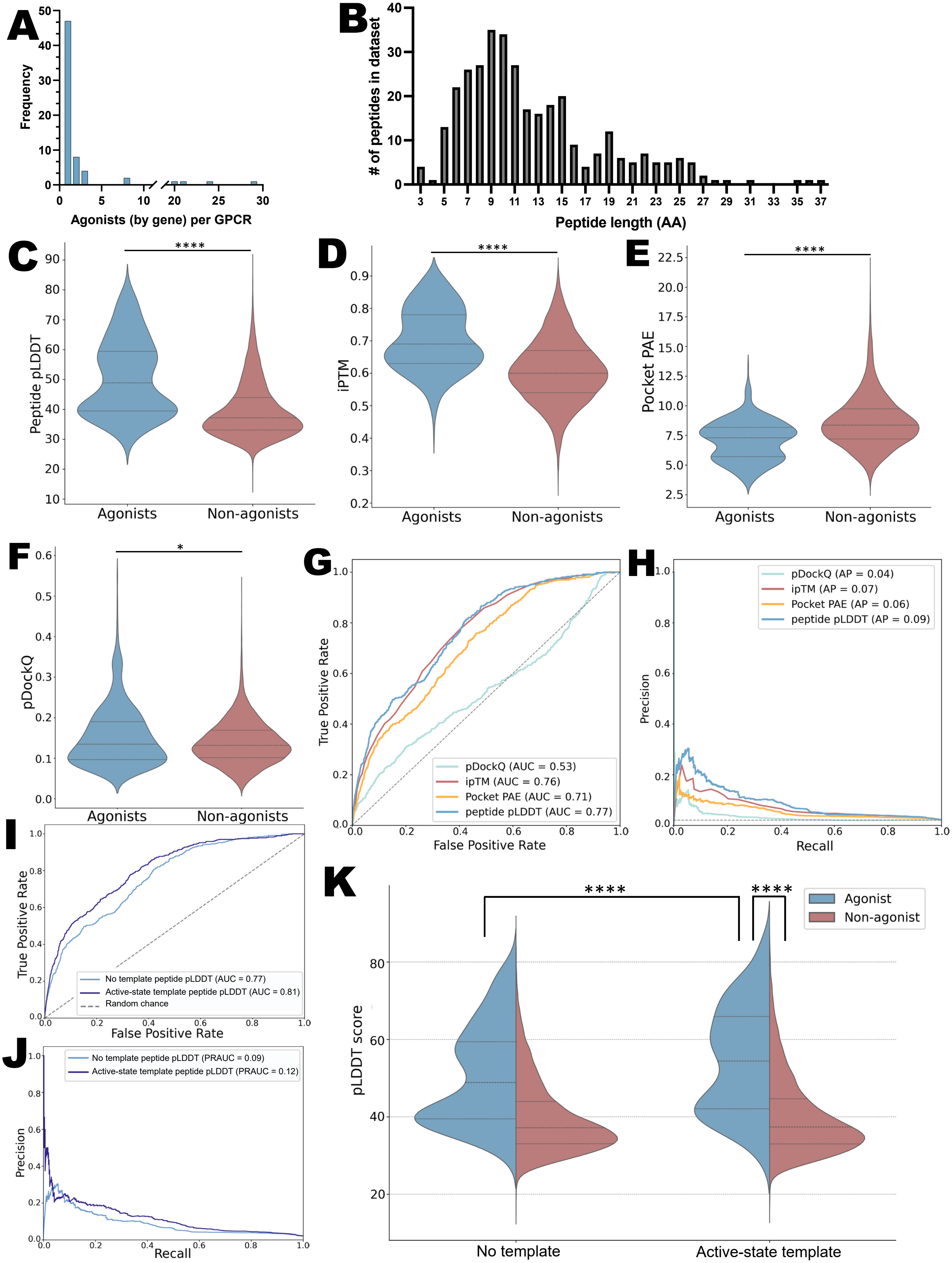

A
Frequency
Agonists (by gene) per GPCR
B
# of peptides in dataset
Peptide length (AA)
C
Peptide pLDDT
Agonists
Non-agonists
****
D
iPTM
Agonists
Non-agonists
****
E
Pocket PAE
Agonists
Non-agonists
****
F
pDockQ
Agonists
Non-agonists
*
G
True Positive Rate
False Positive Rate
pDockQ (AUC = 0.53)
ipTM (AUC = 0.76)
Pocket PAE (AUC = 0.71)
peptide pLDDT (AUC = 0.77)
H
Precision
Recall
pDockQ (AP = 0.04)
ipTM (AP = 0.07)
Pocket PAE (AP = 0.06)
peptide pLDDT (AP = 0.09)
I
True Positive Rate
False Positive Rate
No template peptide pLDDT (AUC = 0.77)
Active-state template peptide pLDDT (AUC = 0.81)
Random chance
J
Precision
Recall
No template peptide pLDDT (PRAUC = 0.09)
Active-state template peptide pLDDT (PRAUC = 0.12)
K
pLDDT score
Agonist
Non-agonist
****
****
No template
Active-state template

Supplemental Figure 1: Evaluation of AF-Multimer confidence metrics for agonist and non-agonist interactions, related to Figure 1 and Figure 2.
(**A**) Frequency distribution of the number of agonists by gene per GPCR in the *C. elegans* dataset. (**B**) Frequency distribution of peptide lengths. (**C-F**) AF2 metrics were compared between agonists (blue, n = 457) and non-agonists (red, n = 21 578). Violin plots show the median and interquartile range (IQR); statistical comparisons were performed using two-sided Mann-Whitney tests. (**C**) Agonist peptide pLDDT is significantly higher than non-agonist values, **** $p < 0.0001$. (**D**) Agonist iPTM is significantly higher than non-agonist values, **** $p < 0.0001$. (**E**) Agonist pocket PAE is significantly higher than non-agonist values, **** $p < 0.0001$. (**F**) Agonist pDockQ is significantly higher than non-agonist values, * $p < 0.05$. (**G**) Receiver operating characteristic (ROC) area under the curve (AUC) and (**H**) Precision-recall (PR) AUC for ipTM (ROC-AUC, 0.756; PR-AUC, 0.072; F1, 0.147; MCC, 0.128), peptide pLDDT (ROC-AUC, 0.766; PR-AUC, 0.089; F1, 0.171; MCC, 0.155), pocket PAE (ROC-AUC, 0.709; PR-AUC, 0.055; F1, 0.122; MCC, 0.107), and pDockQ (ROC-AUC, 0.534; PR-AUC, 0.036; F1, 0.081; MCC, 0.060). (**I**) ROC-AUC and PR-AUC for peptide pLDDT of AF-Multimer structures modelled with active-state templates (ROC-AUC, 0.807; PR-AUC, 0.119; F1, 0.209; MCC, 0.193). No template peptide pLDDT is shown for comparison (same data from Panels G and H). (**K**) Peptide pLDDT of agonists is significantly increased by modelling with active-state templates compared to modelling without templates. When modelling with active-state templates, peptide pLDDT is significantly greater for agonist compared to non-agonist interactions. Statistical comparisons were carried out by two-sided Mann–Whitney U test with Bonferonni correction for multiple comparisons, **** $p < 0.0001$. No template data is the same as in panel C.

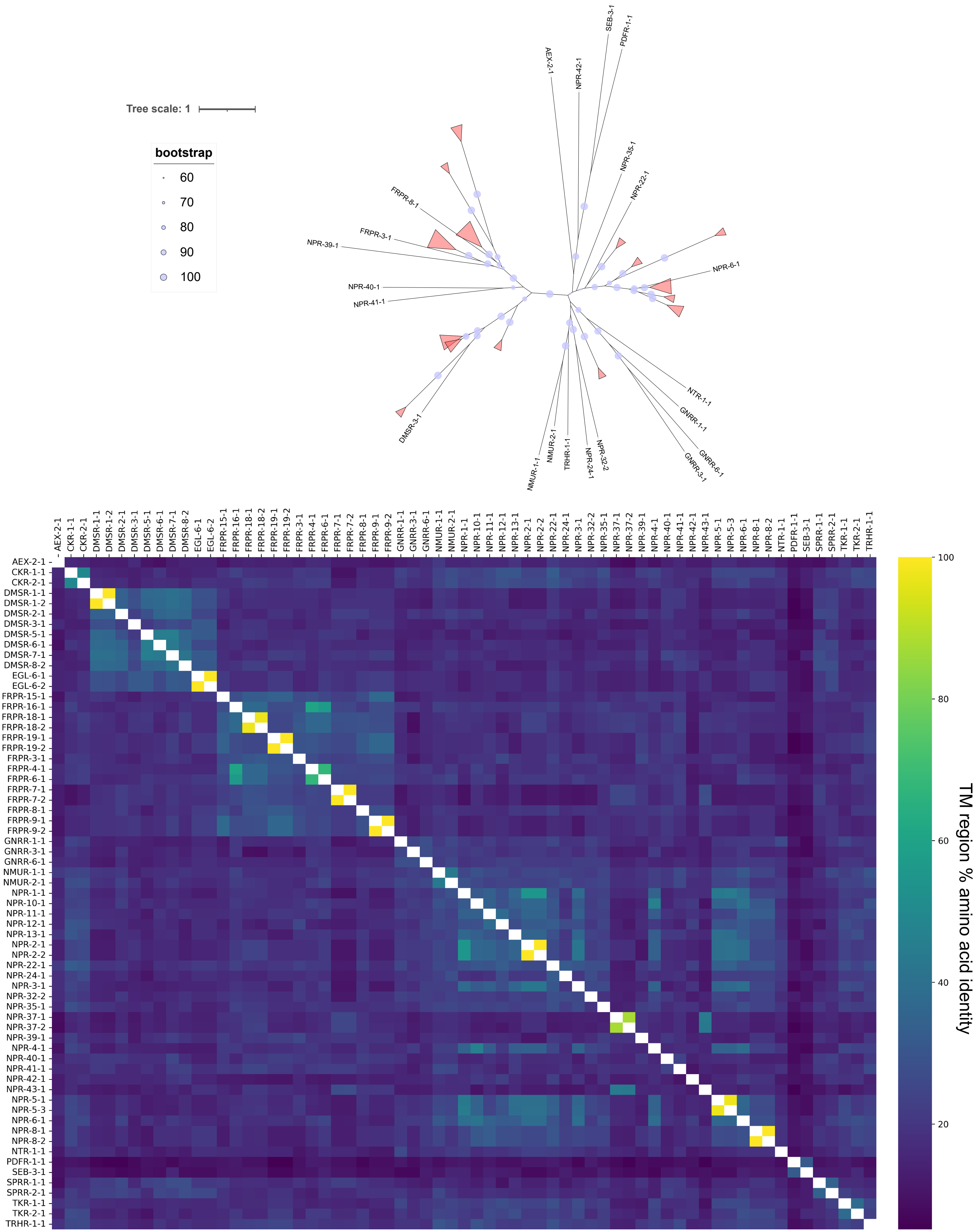
Tree scale: 1
bootstrap
60
70
80
90
100
SEB-3-1
PDFR-1-1
AEX-2-1
NPR-42-1
NPR-35-1
NPR-22-1
FRPR-8-1
FRPR-3-1
NPR-39-1
NPR-6-1
NPR-40-1
NPR-41-1
NTR-1-1
GNRR-1-1
GNRR-6-1
GNRR-3-1
DMSR-3-1
NMUR-2-1
NMUR-1-1
TRHR-1-1
NPR-24-1
NPR-32-2
AEX-2-1
CKR-1-1
CKR-2-1
DMSR-1-1
DMSR-1-2
DMSR-2-1
DMSR-3-1
DMSR-5-1
DMSR-6-1
DMSR-7-1
DMSR-8-2
EGL-6-1
EGL-6-2
FRPR-15-1
FRPR-16-1
FRPR-18-1
FRPR-18-2
FRPR-19-1
FRPR-19-2
FRPR-3-1
FRPR-4-1
FRPR-6-1
FRPR-7-1
FRPR-7-2
FRPR-8-1
FRPR-9-1
FRPR-9-2
GNRR-1-1
GNRR-3-1
GNRR-6-1
NMUR-1-1
NMUR-2-1
NPR-1-1
NPR-10-1
NPR-11-1
NPR-12-1
NPR-13-1
NPR-2-1
NPR-2-2
NPR-22-1
NPR-24-1
NPR-3-1
NPR-32-2
NPR-35-1
NPR-37-1
NPR-37-2
NPR-39-1
NPR-4-1
NPR-40-1
NPR-41-1
NPR-42-1
NPR-43-1
NPR-5-1
NPR-5-3
NPR-6-1
NPR-8-1
NPR-8-2
NTR-1-1
PDFR-1-1
SEB-3-1
SPRR-1-1
SPRR-2-1
TKR-1-1
TKR-2-1
TRHR-1-1
100
80
60
40
20
TM region % amino acid identity

Supplemental Figure 2: Phylogenetic relationships of transmembrane (TM) bundle domains of *C. elegans* GPCRs in the training dataset, related to Figure 3. (**A**) Phylogeny inferred from aligned and trimmed TM bundle regions. Clusters of receptors separated by <1.48 substitutions per site are shown as red triangles; triangle size corresponds to the number of GPCRs within each cluster. These clusters were kept intact when assigning GPCRs to cross-validation folds, and the members of each cluster are listed in **Supplemental Data 1**. Branch support values were estimated using ultrafast bootstrap analysis. (**B**) Heatmap of pairwise amino acid percent identity computed from the aligned and trimmed TM bundle domains.

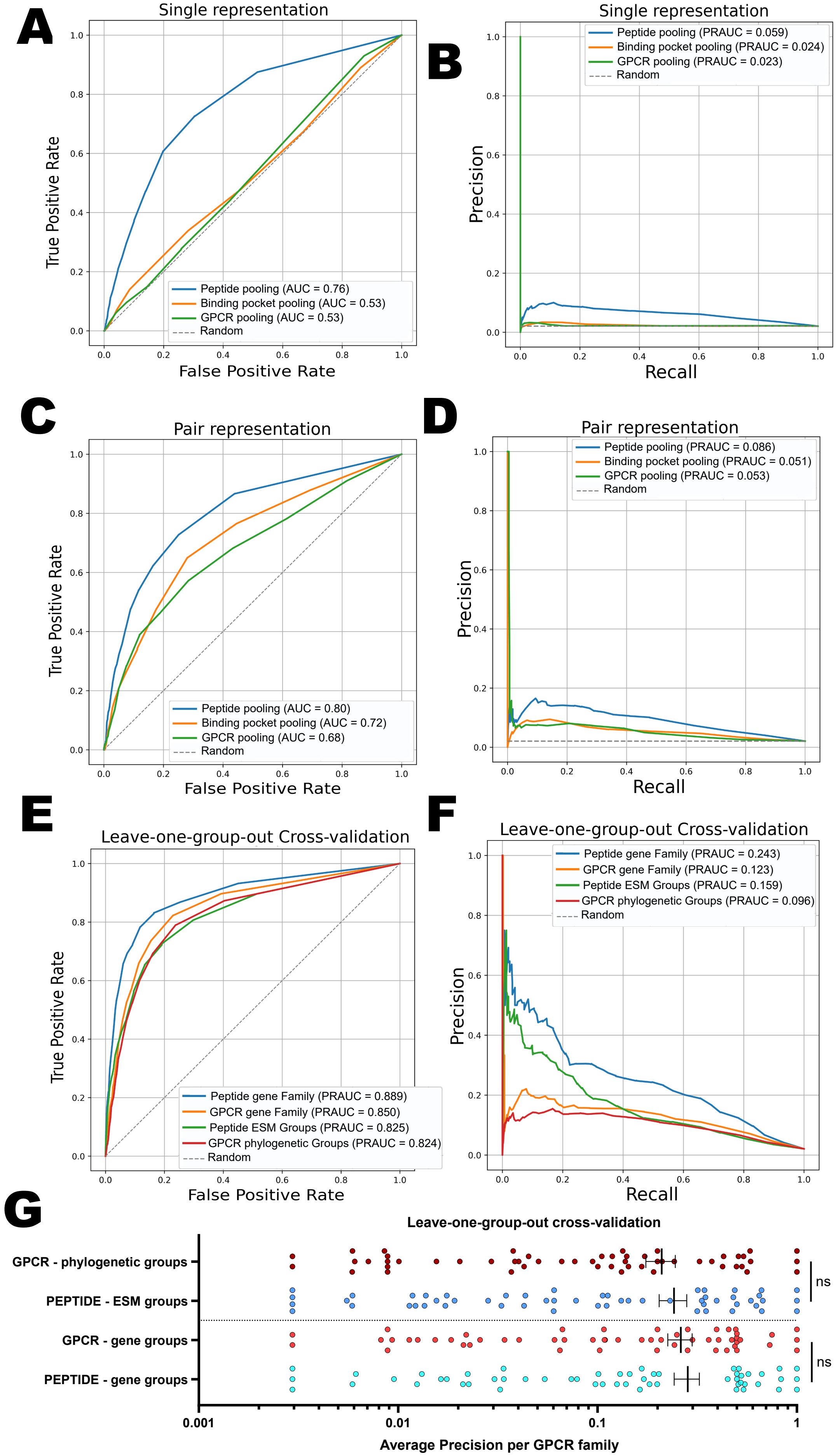

A
Single representation
True Positive Rate
False Positive Rate
Peptide pooling (AUC = 0.76)
Binding pocket pooling (AUC = 0.53)
GPCR pooling (AUC = 0.53)
Random
B
Single representation
Precision
Recall
Peptide pooling (PRAUC = 0.059)
Binding pocket pooling (PRAUC = 0.024)
GPCR pooling (PRAUC = 0.023)
Random
C
Pair representation
True Positive Rate
False Positive Rate
Peptide pooling (AUC = 0.80)
Binding pocket pooling (AUC = 0.72)
GPCR pooling (AUC = 0.68)
Random
D
Pair representation
Precision
Recall
Peptide pooling (PRAUC = 0.086)
Binding pocket pooling (PRAUC = 0.051)
GPCR pooling (PRAUC = 0.053)
Random
E
Leave-one-group-out Cross-validation
True Positive Rate
False Positive Rate
Peptide gene Family (PRAUC = 0.889)
GPCR gene Family (PRAUC = 0.850)
Peptide ESM Groups (PRAUC = 0.825)
GPCR phylogenetic Groups (PRAUC = 0.824)
Random
F
Leave-one-group-out Cross-validation
Precision
Recall
Peptide gene Family (PRAUC = 0.243)
GPCR gene Family (PRAUC = 0.123)
Peptide ESM Groups (PRAUC = 0.159)
GPCR phylogenetic Groups (PRAUC = 0.096)
Random
G
Leave-one-group-out cross-validation
GPCR - phylogenetic groups
PEPTIDE - ESM groups
GPCR - gene groups
PEPTIDE - gene groups
ns
ns
0.001
0.01
0.1
1
Average Precision per GPCR family

Supplemental Figure 3: Evaluation of random forest models trained on AF2-multimer single and pair representations, related to Figure 3.

(**A, C**) ROC-AUC and (**B, D**) PR-AUC graphs for the data presented in Figure 3B-C. For the single representations (**A-B**), peptide pooling achieved ROC-AUC = 0.763, PR-AUC = 0.059, F1 = 0.123, MCC = 0.117; binding pocket pooling achieved ROC-AUC = 0.527, PR-AUC = 0.024, F1 = 0.054, MCC = 0.027; GPCR pooling achieved ROC-AUC = 0.527, PR-AUC = 0.023, F1 = 0.043, MCC = 0.024. For the pair representations (**C-D**), peptide pooling achieved ROC-AUC = 0.798, PR-AUC = 0.086, F1 = 0.178, MCC = 0.166; binding pocket pooling achieved ROC-AUC = 0.716, PR-AUC = 0.051, F1 = 0.117, MCC = 0.097; GPCR pooling achieved ROCAUC = 0.677, PR-AUC = 0.053, F1 = 0.117, MCC = 0.109. (**E**) ROC-AUC, (**F**) PR-AUC, and (**G**) average precision (AP) per GPCR gene family for leave-one-group-out (LOGO) cross-validation with groups determined by GPCR gene family (55 groups) (ROC-AUC, 0.850; PR-AUC, 0.123; F1, 0.222; MCC, 0.227; mAP, 0.262 ± 0.036) or peptide gene family (93 groups) (ROC-AUC, 0.888; PR-AUC, 0.243; F1, 0.327; MCC, 0.327; mAP, 0.283 ± 0.041), and 10-fold cross validation with folds containing phylogenetically similar GPCRs (Table 1) (ROC-AUC, 0.824; PR-AUC, 0.096; F1, 0.197; MCC, 0.190; mAP, 0.210 ± 0.035), or peptides clustered by ESM embedding similarity (Supplemental Table 1) (ROC-AUC, 0.825; PR-AUC, 0.159; F1, 0.247; MCC, 0.232; mAP, 0.242 ± 0.038), using features derived from the pair representation peptide subregion. Error bars indicate mean ± SEM. Differences in mAP across clustering strategies was compared using a Friedman test with Dunn's post hoc comparisons (Friedman $p = 0.121$; all pairwise comparisons ns, adjusted $p \geq 0.295$).

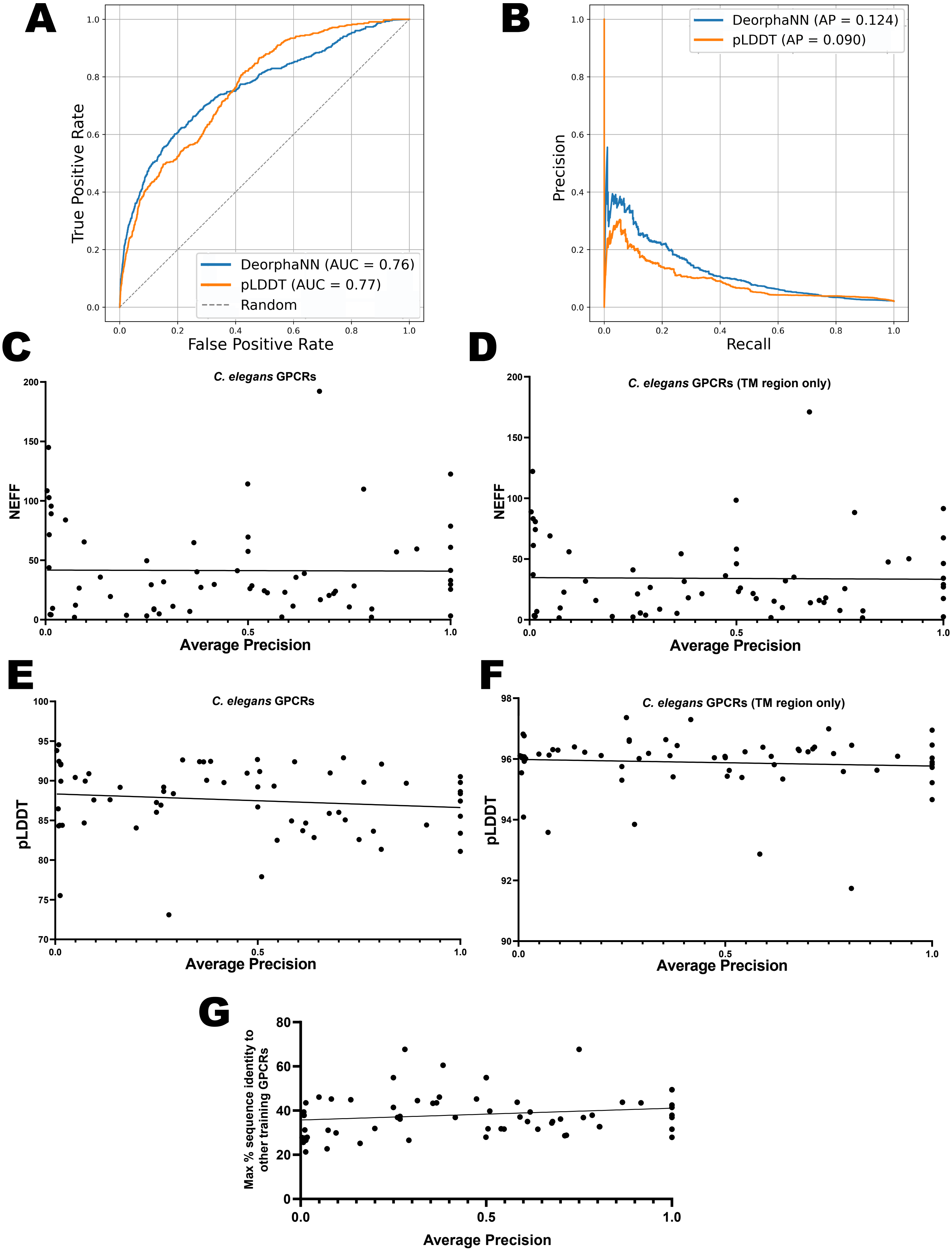

A
True Positive Rate
False Positive Rate
DeorphaNN (AUC = 0.76)
pLDDT (AUC = 0.77)
Random
B
Precision
Recall
DeorphaNN (AP = 0.124)
pLDDT (AP = 0.090)
C
C. elegans GPCRs
NEFF
Average Precision
D
C. elegans GPCRs (TM region only)
NEFF
Average Precision
E
C. elegans GPCRs
pLDDT
Average Precision
F
C. elegans GPCRs (TM region only)
pLDDT
Average Precision
G
Max % sequence identity to other training GPCRs
Average Precision

Supplemental Figure 4: Performance of DeorphaNN does not correlate with the MSA Normalized Effective Number of Sequences (NEFF), pLDDT, or transmembrane (TM) sequence identity, related to Figure 4.

(**A-B**) Performance of DeorphaNN and peptide pLDDT. (**A**) ROC-AUC and (**B**) PR-AUC. DeorphaNN: ROC-AUC, 0.763; PR-AUC, 0.124; F1, 0.217; MCC, 0.201. Peptide pLDDT: ROC-AUC, 0.766; PR-AUC, 0.089; F1, 0.171; MCC, 0.155, same data as in Supplemental Figure 1G-H. (**C-G**) Per-GPCR AP from DeorphaNN was correlated against evolutionary information, modelling confidence scores, and similarity to GPCRs in the training set. Correlation of DeorphaNN AP against (**C**) NEFF of the entire GPCR, (**D**) NEFF averaged across the GPCR TM region only, (**E**) GPCR average pLDDT, (**F**) GPCR TM region average pLDDT, and (**G**) maximum percent sequence identity of the GPCR (TM region) to any other training GPCR (exclusing isoforms from the same GPCR gene). Correlations were assessed using two-tailed Pearson correlation. No significant correlations were observed in any comparison (**C**: $r = –0.008$, $p = 0.95$; **D**: $r = –0.014$, $p = 0.91$; **E**: $r = –0.13$, $p = 0.29$; **F**: $r = –0.078$, $p = 0.54$; **G**: $r = 0.181$, $p = 0.15$; $n = 65$).

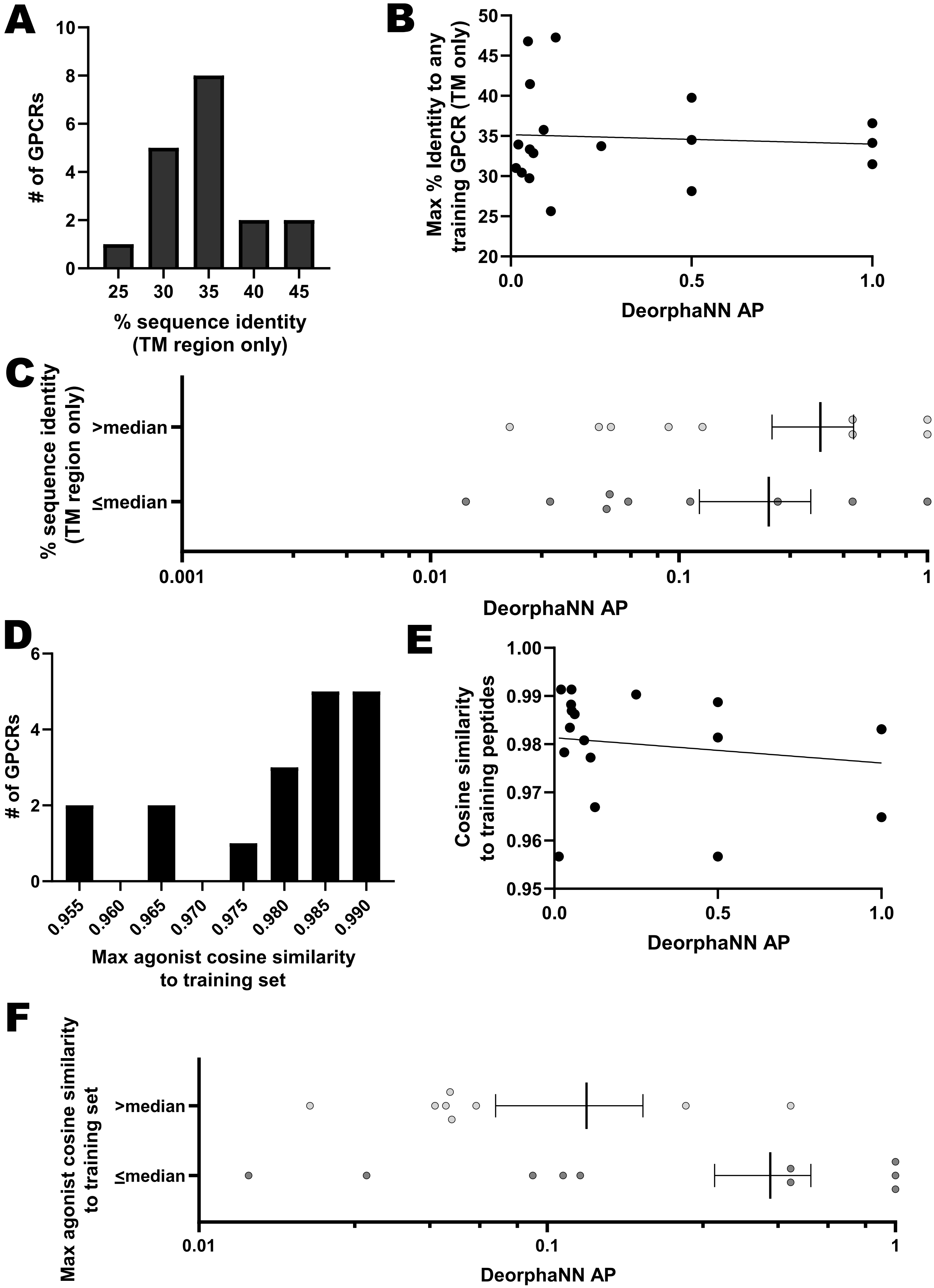
A
# of GPCRs
0
2
4
6
8
10
25
30
35
40
45
% sequence identity
(TM region only)
B
Max % Identity to any
training GPCR (TM only)
20
25
30
35
40
45
50
0.0
0.5
1.0
DeorphaNN AP
C
% sequence identity
(TM region only)
>median
≤median
0.001
0.01
0.1
1
DeorphaNN AP
D
# of GPCRs
0
2
4
6
0.955
0.960
0.965
0.970
0.975
0.980
0.985
0.990
Max agonist cosine similarity
to training set
E
Cosine similarity
to training peptides
0.95
0.96
0.97
0.98
0.99
1.00
0.0
0.5
1.0
DeorphaNN AP
F
Max agonist cosine similarity
to training set
>median
≤median
0.01
0.1
1
DeorphaNN AP

Supplemental Figure 5: Performance of DeorphaNN on a *Platynereis dumerilli* dataset does not correlate with TM sequence identity or peptide similarity, related to Figure 5.

(**A**) Frequency distribution of *Platynereis* GPCRs in 5% sequence identity bins of the TM regions relative to the nearest *C. elegans* GPCR in the training set. (**B**) Maximum percent sequence identity of the TM regions to any *C. elegans* GPCR in the training set is plotted against DeorphaNN AP per *Platynereis* GPCR, showing no significant correlation between AP and sequence identity (Pearson $r = -0.073$, $P = 0.77$, $n = 18$). (**C**) AP stratified by the median of TM percent sequence identity (≤median vs >median). No significant difference was observed between groups (≤median: $n = 9$, mean 0.23 ± 0.11 SEM; >median: $n = 9$, mean 0.37 ± 0.13 SEM; Mann–Whitney U test, $p = 0.43$). (**D**) Frequency distribution of *Platynereis* GPCRs binned by average maximum agonist cosine similarity to peptides in the training set. (**E**) Average maximum cosine similarity of agonists to *C. elegans* peptides in the training set plotted against the AP of DeorphaNN scores for each *Platynereis* GPCR, showing no significant correlation between AP and peptide similarity (Pearson $r = -0.1698$, $P = 0.50$, $n = 18$). (**F**) DeorphaNN AP per *Platynereis* GPCR stratified around the median of the average maximum cosine similarity of agonists to training peptides. No significant difference was observed between bins (≤median: $n = 10$, mean, 0.43 ± 0.13 SEM; >median: $n = 8$, mean, 0.13 ± 0.06 SEM; Mann–Whitney U test, $p = 0.12$).

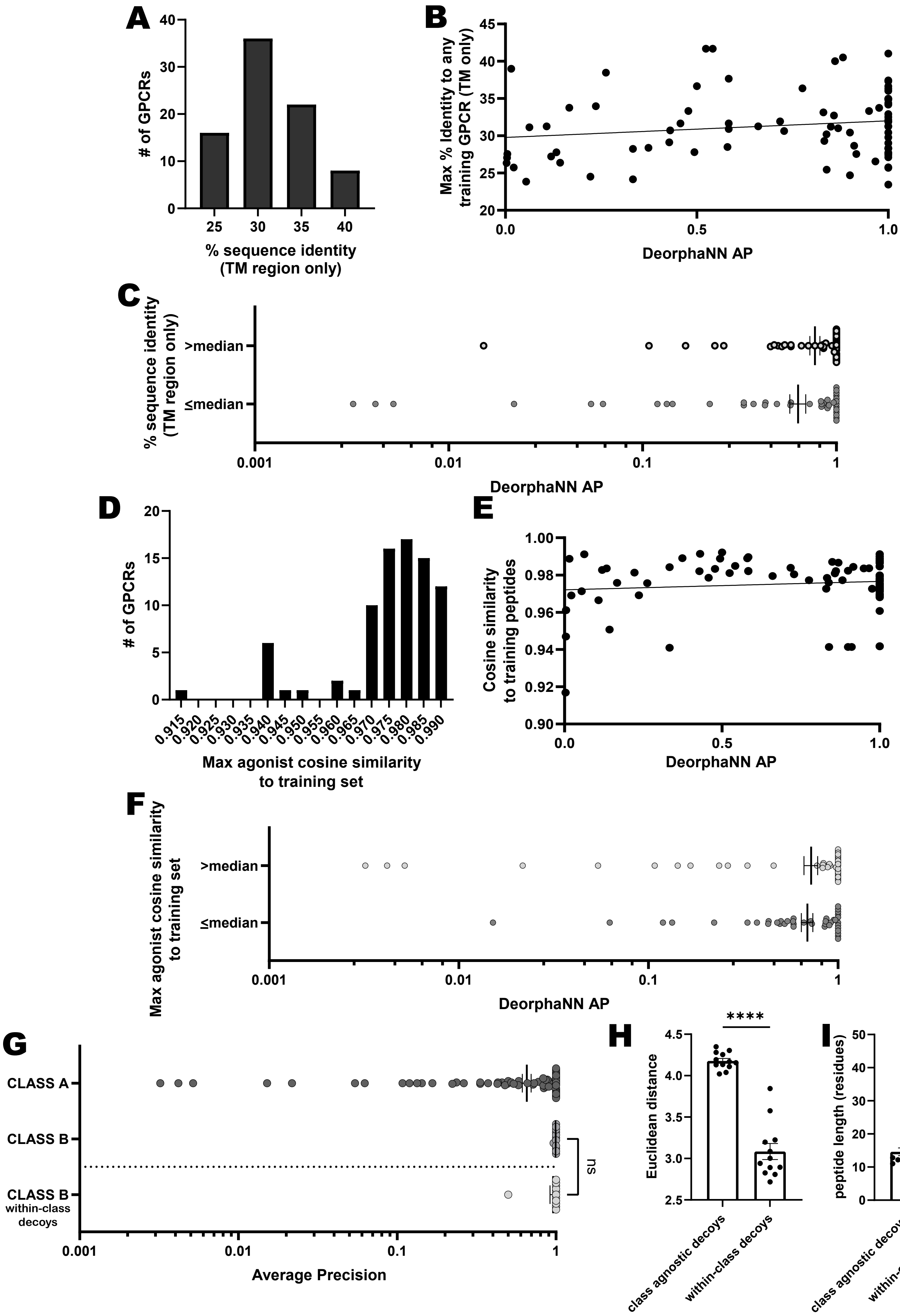

A
# of GPCRs
% sequence identity (TM region only)
B
Max % Identity to any training GPCR (TM only)
DeorphaNN AP
C
% sequence identity (TM region only)
>median
≤median
DeorphaNN AP
D
# of GPCRs
Max agonist cosine similarity to training set
E
Cosine similarity to training peptides
DeorphaNN AP
F
Max agonist cosine similarity to training set
>median
≤median
DeorphaNN AP
G
CLASS A
CLASS B
CLASS B within-class decoys
ns
Average Precision
H
Euclidean distance
****
class agnostic decoys
within-class decoys
I
peptide length (residues)
****
class agnostic decoys
within-class decoys

Supplemental Figure 6: Performance of DeorphaNN on a human dataset does not correlate with TM sequence identity, peptide similarity or peptide class, related to Figure 5.

(**A**) Frequency distribution of human GPCRs in 5% sequence identity bins of the TM regions relative to the nearest *C. elegans* GPCR in the training set. (**B**) Maximum percent sequence identity of the TM regions to any *C. elegans* GPCR in the training set is plotted against DeorphaNN AP per human GPCR, showing no significant correlation between AP and sequence identity (Pearson $r = 0.17$, $P = 0.12$, $n = 82$). (**C**) AP of DeorphaNN scores per human GPCR stratified by median TM percent sequence identity. No significant difference was observed between groups (≤median: $n = 41$, mean, 0.63 ± 0.06 SEM; >median: $n = 41$, mean, 0.78 ± 0.05 SEM; Mann–Whitney U test, $p = 0.08$). (**D**) Frequency distribution of human GPCRs binned by average maximum agonist cosine similarity to peptides in the training set. (**E**) Average maximum cosine similarity of agonists to peptides in the training set plotted against DeorphaNN AP per human GPCR, showing no significant correlation between AP and peptide similarity (Pearson $r = 0.1087$, $P = 0.33$, $n = 82$). (**F**) DeorphaNN AP per human GPCR stratified by the median average maximum cosine similarity of agonists to training peptides. No significant difference was observed between groups (≤median: $n = 41$, mean, 0.69 ± 0.05 SEM; >median: $n = 41$, mean, 0.72 ± 0.06 SEM; Mann–Whitney U test, $p = 0.31$). (**G**) DeorphaNN AP split by Class A and B GPCRs in the human validation dataset (same data as Figure 5C, using class agnostic peptide decoys). AP was recalculated for Class B GPCRs using within-class decoys. Class A GPCRs, mAP = 0.655 ± 0.04 SEM; Class B GPCRs, mAP = 0.997 ± 0.003 SEM; Class B GPCRs (within-class decoys), mAP = 0.958 ± 0.04 SEM. AP between class B GPCRs in complex with class agnostic decoy peptides, or within-class decoy peptides only was not significant (Wilcoxin matched-pairs signed rank test; $p > 0.999$). Class A GPCRs are shown for descriptive purposes. (**H**) Mean Euclidean distance between decoy peptides and cognate agonist(s) in ESM-2 embedding space, computed per GPCR. Within-class decoys for Class B GPCRs show significantly reduced distance to cognate agonists compared with class agnostic decoys (Paired two-tailed t-test, **** $p < 0.0001$). (**I**) Mean peptide length of decoy peptides per GPCR. Class agnostic decoys are significantly longer than within-class decoys (Paired two-tailed t-test, **** $p < 0.0001$).

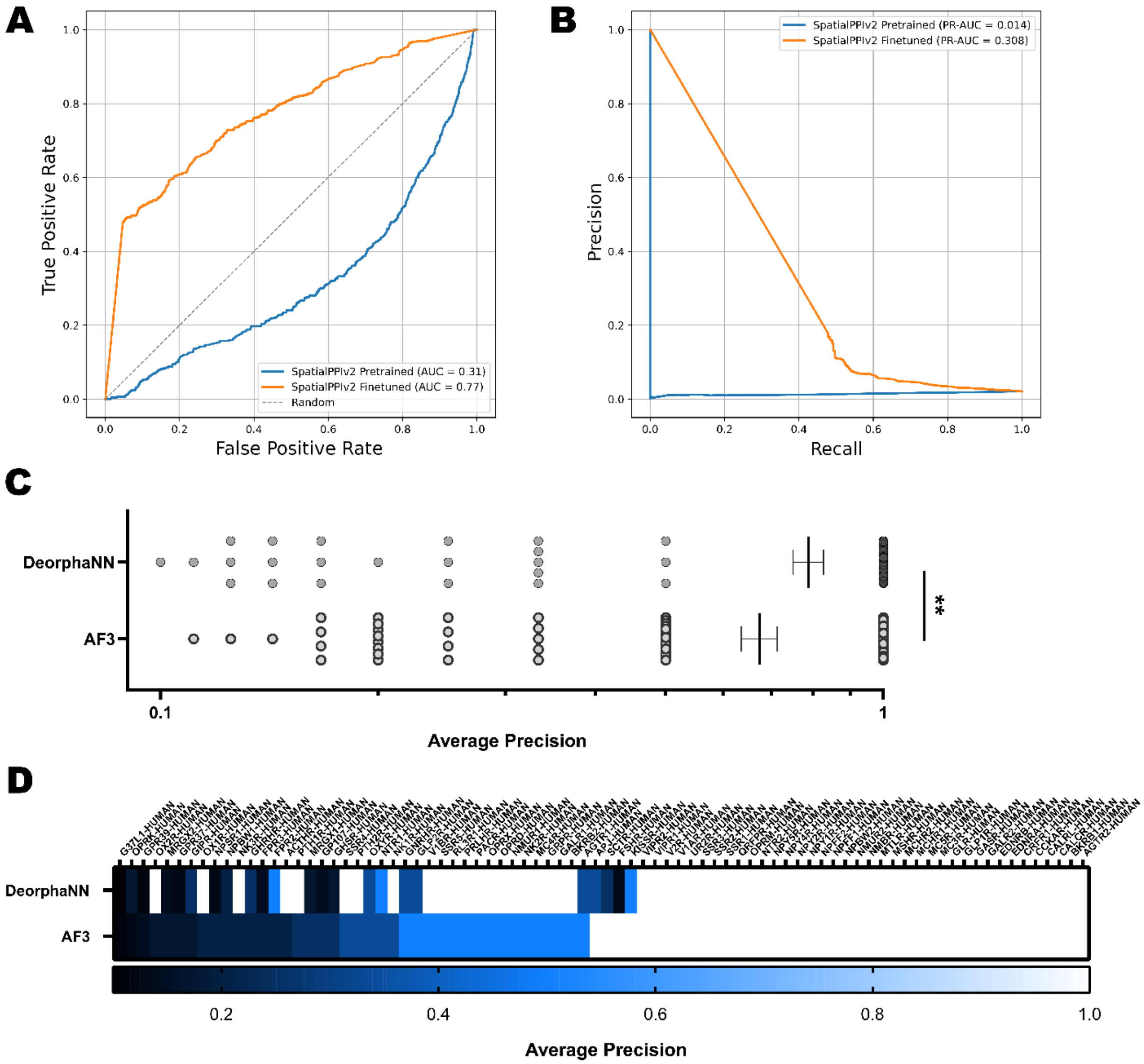

Supplemental Figure 7: Finetuning of SpatialPPIv2 on the C. elegans dataset and performance comparison of DeorphaNN to AlphaFold3, related to Figure 5.

SpatialPPIv2 was fine-tuned on the *C. elegans* dataset. (**A**) ROC-AUC and (**B**) PR-AUC graphs of the pretrained (not fine-tuned; ROC-AUC, 0.314; PR-AUC, 0.014; F1, 0.041; MCC, 0.013) and fine-tuned (ROC-AUC, 0.771; PR-AUC, 0.111; F1, 0.258; MCC, 0.267) models are shown. (**C**) DeorphaNN outperforms AlphaFold3 in prioritizing peptide agonists for human GPCRs. AF3 interaction scores were as previously published[1], and DeorphaNN was evaluated on identical GPCR–peptide pairs to allow a direct method-to-method comparison. DeorphaNN mAP = 0.79; AF3 mAP = 0.67 (Wilcoxon matched-pairs signed rank test, ** $p < 0.01$). (**D**) Heatmap for the per-GPCR AP values from (A), illustrating that DeorphaNN prioritizes agonists missed by AF3.

Supplemental Table 1: Cross-validation folds grouped by GPCR phylogenetic similarity, related to Figure 4

Fold	GPCRs	# agonists in set	Fraction of GPCRs with at least one agonist in top 5 by rank	mAP (mean AP ± SEM)	ROCAUC	PRAUC
1	FRPR-16-1, FRPR-18-1, FRPR-18-2, FRPR-4-1, FRPR-6-1, NPR-22-1, NMUR-2-1	32	6/7	0.423 ± 0.099	0.882	0.334
2	AEX-2-1, DMSR-5-1, DMSR-6-1, DMSR-7-1, DMSR-8-2, NPR-32-2	81	3/6	0.285 ± 0.159	0.850	0.147
3	FRPR-7-1, FRPR-7-2, NPR-6-1, GNRR-3-1, EGL-6-1, EGL-6-2	58	5/6	0.456 ± 0.113	0.925	0.357
4	TKR-1-1, TKR-2-1, DMSR-1-1, DMSR-1-2, DMSR-2-1, NPR-40-1, GNRR-6-1	108	3/7	0.298 ± 0.122	0.828	0.246
5	NPR-42-1, FRPR-15-1, FRPR-19-1, FRPR-19-2, FRPR-9-1, FRPR-9-2, NMUR-1-1	13	6/7	0.440 ± 0.118	0.894	0.108
6	NPR-8-1, NPR-8-2, NPR-24-1, NPR-37-1, NPR-37-2, NPR-43-1, FRPR-3-1	22	6/7	0.598 ± 0.135	0.874	0.619
7	FRPR-8-1, GNRR-1-1, SPRR-1-1, SPRR-2-1, NPR-11-1, NPR-12-1	60	6/6	0.723 ± 0.079	0.857	0.266
8	NPR-41-1, NPR-1-1, NPR-2-1, NPR-2-2, NPR-3-1, PDFR-1-1, NTR-1-1	18	5/7	0.450 ± 0.119	0.660	0.137
9	TRHR-1-1, NPR-35-1, NPR-13-1, NPR-5-1, NPR-5-3, SEB-3-1	28	4/6	0.379 ± 0.144	0.930	0.269
10	DMSR-3-1, NPR-10-1, NPR-4-1, NPR-39-1, CKR-1-1, CKR-2-1	37	3/6	0.417 ± 0.192	0.594	0.100
		457	47/65	0.452 ± 0.042	0.829 ± 0.035	0.258 ± 0.050

Supplemental Table 2: **Leave-one-peptide-family out applied to DeorphaNN for highest 10 peptide families by agonist interactions, related to Figure 4**

Peptide Family	# peptides	# agonist interactions	ROC-AUC	PR-AUC
FLP-1	10	46	0.823	0.227
FLP-18	10	42	0.598	0.075
FLP-13	7	35	0.938	0.387
FLP-17	4	21	0.983	0.848
FLP-3	11	21	0.699	0.067
FLP-8	2	18	0.936	0.661
FLP-27	6	16	0.837	0.573
FLP-11	4	15	0.821	0.286
FLP-5	3	15	0.962	0.704
NLP-13	7	14	0.617	0.398
			**0.821 ± 0.044**	**0.422 ± 0.080**

References:

1. Hoegen Dijkhof, L. R., Rönkkö, T. K. E., Von Vegesack, H. C., Lenzing, J. & Hauser, A. S. Deep learning in GPCR drug discovery: benchmarking the path to accurate peptide binding. *Briefings in Bioinformatics* **26**, bbaf186 (2025).
